# CRISPR-Cas9-mediated upregulation of utrophin ameliorates Duchenne muscular dystrophy

**DOI:** 10.1016/j.ymthe.2026.03.025

**Published:** 2026-03-24

**Authors:** Maëlle Ralu, Simon Guiraud, Sumitava Dastidar, Paola Galbiati, Emilia Sadaoui, Fetta Mazed, Fatima Amor, Anne de Cian, Isabelle Richard, Kamel Mamchaoui, Giuseppe Ronzitti, Francesco Saverio Tedesco, Mario Amendola

**Affiliations:** 1 Genethon, 91000 Evry, France; 2 Université Paris-Saclay, University of Evry, Inserm, Genethon, Integrare Research Unit UMR_S951, 91000 Evry-Courcouronnes, France; 3 Department of Cell and Developmental Biology, University College London, WC1E 6BT London, UK; 4 The Francis Crick Institute, NW1 1AT London, UK; 5 INSERM U1154, CNRS UMR7196, Museum National D'Histoire Naturelle, 75015 Paris, France; 6 Sorbonne Université, Inserm, Institut de Myologie, Centre de Recherche en Myologie, 75013 Paris, France; 7 Dubowitz Neuromuscular Centre, UCL Great Ormond Street Institute of Child Health and Great Ormond Street Hospital for Children, WC1N 1EH London, UK; 8 Department of Clinical and Experimental Medicine, University of Foggia, 71122 Foggia, Italy

**Keywords:** DMD, utrophin, CRISPR-Cas9, rAAV, gene therapy, genome editing, miRNA Let-7c

## Abstract

Duchenne muscular dystrophy (DMD) is a lethal neuromuscular disorder caused by loss of dystrophin. Upregulating utrophin, a dystrophin paralog, is a promising gene therapy approach. Here, we present a CRISPR-Cas9-based strategy to enhance utrophin expression by disrupting repressor binding sites. Using a Cas9/guide RNA (gRNA) ribonucleoprotein complex, we disrupted several such sites in DMD myoblasts and identified microRNA Let-7c binding site as effective in relieving repression of the *UTRN* gene. Interestingly, Cas9-generated insertions or deletions (indels) were as effective as the complete removal of Let-7c binding site in upregulating UTRN expression, with minimal off-target effects. In a three-dimensional tissue-engineered human skeletal muscle model of DMD, this editing strategy resulted in significant utrophin upregulation and functional improvements of calcium dysregulation and muscle contraction. Finally, in *mdx* mice, local or systemic delivery of recombinant adeno-associated viruses encoding Cas9 and gRNA targeting the Let-7c binding site resulted in utrophin upregulation and amelioration of muscle histopathology and function. These findings provide the foundations for a mutation-independent, potentially universal gene-editing therapeutic strategy for DMD.

## Graphical abstract

**Figure:**
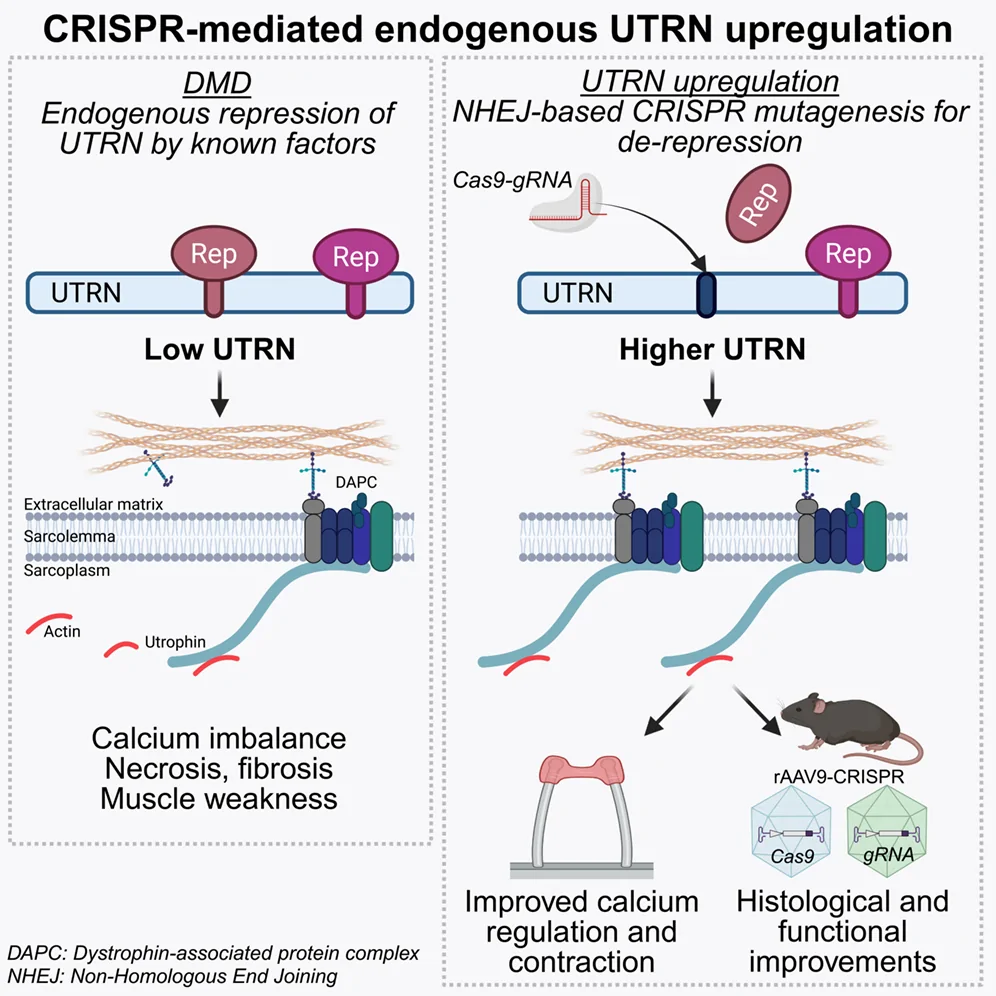

## Teaser

Amendola and colleagues demonstrated that targeted CRISPR-Cas9-mediated disruption of a single microRNA repressor binding site durably upregulates endogenous utrophin in human and murine DMD models. This mutation-independent strategy improves muscle structure and function, highlighting a promising genome-editing approach broadly applicable to patients with DMD.

## Introduction

Duchenne muscular dystrophy (DMD; OMIM: 310200)^1^^,^^2^ is an X-linked, progressive, and lethal neuromuscular disorder affecting 1 in 5,000 newborn males.^3^^,^^4^ This disease is caused by pathogenic variants in the *DMD* gene, which encodes a large sub-sarcolemmal protein called dystrophin.^5^ Dystrophin creates a stable link between the extracellular matrix and the cytoskeleton, providing muscle fibers with strength, flexibility, and stability and allowing them to cope with repeated cycles of muscle contraction and relaxation.^6^^,^^7^ In DMD, dystrophin deficiency renders the sarcolemma highly susceptible to contraction-induced injuries, causing progressive proximal-to-distal muscle degeneration and replacement of contractile material with adipose and fibrotic tissues. DMD patients manifest the first onset of symptoms in early childhood, generally lose ambulation by the age of 12 years, and develop respiratory and cardiac failure leading to premature death by their early thirties.^8^^,^^9^^,^^10^^,^^11^

Despite progress in symptomatic management of the disease,^12^^,^^13^^,^^14^ efficient treatment options remain limited for DMD. Efforts in developing strategies to recover dystrophin expression have led to the approval of several products based on exon-skipping^15^^,^^16^^,^^17^^,^^18^ or gene transfer.^19^ However, these approaches are often mutation specific or rely on truncated proteins, addressing only specific subsets of DMD patients and providing limited clinical benefits, respectively. In addition, dystrophin-directed immune response was observed in some patients,^20^^,^^21^^,^^22^ questioning the safety and longevity of such strategies. An alternative therapeutic approach, potentially suitable to all DMD patients irrespective of their genetic defect, consists in upregulating utrophin (UTRN), a structural and functional paralog of dystrophin.^23^ Specifically, its longest isoform—UTRN A—is the muscle-specific paralog of dystrophin Dp427. Although it differs from dystrophin in its mode of interaction with microtubules^24^ and actin filaments,^25^^,^^26^ and it cannot anchor the neuronal nitric oxide synthase (nNOS),^27^ UTRN’s sarcolemmal organization closely resembles that of dystrophin.^6^^,^^28^ In healthy conditions, UTRN is ubiquitously localized at the sarcolemma *in utero* and then progressively replaced by dystrophin.^29^^,^^30^ In adult muscles, it is restricted to the neuromuscular and myotendinous junctions^31^^,^^32^^,^^33^ as well as the sarcolemma of regenerating myofibers.^34^^,^^35^ Many studies have demonstrated that UTRN is naturally upregulated in the muscles of DMD patients^36^^,^^37^^,^^38^^,^^39^ and some studies have suggested a correlation between the level of UTRN and the severity of the DMD phenotype.^37^^,^^40^^,^^41^ Interestingly, UTRN can act as a surrogate to compensate for the lack of dystrophin in DMD mouse^42^^,^^43^^,^^44^^,^^45^ and dog^46^^,^^47^ models. In addition, UTRN has been shown to be non-toxic^48^ and to elicit a milder immune response compared to dystrophin.^47^^,^^49^ While a 1.5-fold increase of UTRN results in a therapeutic benefit, a 3- to 4-fold overexpression prevents the dystrophic pathology.^42^^,^^50^ Several post-natal repressors of UTRN have been identified, such as the transcriptional Ets-2 repressor factor (ERF)^51^ and several microRNAs (miRs) such as miR-150, -296-5p, -133b, -196b, or Let-7c, able to interact with its 3′ untranslated region (UTR).^52^ In particular, previous studies by Khurana’s group demonstrated that antisense oligonucleotides can efficiently mask the Let-7c binding site (BS) and upregulate UTRN while providing functional and histological benefits *in vivo*.^53^^,^^54^ However, this approach only provides transient effects and thus requires repeated administrations.

Recently, the CRISPR-Cas system has emerged as a revolutionary gene-editing tool to permanently and precisely edit any DNA locus.^55^^,^^56^ Several groups used CRISPR-Cas9 to correct and restore dystrophin expression in immortalized DMD patient cells,^57^ as well as in murine^58^^,^^59^^,^^60^ and canine^61^ models of DMD, and to upregulate UTRN by deleting a cluster of repressor BS^62^ or by inducing its transcriptional activation.^63^^,^^64^^,^^65^ In the present study, we used the CRISPR-Cas9 tool to precisely disrupt the BS of known UTRN repressors at the DNA level to permanently upregulate UTRN with a single guide RNA (gRNA). We showed that minimal miR-Let-7c BS disruption induces significant UTRN upregulation in murine and human myoblasts as well as myotubes and provides functional benefits in three-dimensional (3D) tissue-engineered human DMD skeletal muscles. Finally, via local or systemic injection of adeno-associated viruses (AAVs) coding Cas9 and gRNA in *mdx* mice, we demonstrated that our system could upregulate UTRN and ameliorate muscle histopathological and functional phenotypes *in vivo*. In contrast to existing antisense oligonucleotide approaches, our CRISPR-based strategy enables a one-time, permanent disruption of the BS, thereby reducing treatment burden and improving durability. These data demonstrate the *in vitro* and *in vivo* potential of our CRISPR-Cas9 strategy to upregulate UTRN expression as a universal therapeutic strategy for DMD patients.

## Results

### Evaluation of gRNAs targeting repressor BSs for UTRN upregulation in human DMD myoblasts

**Figure 1:**
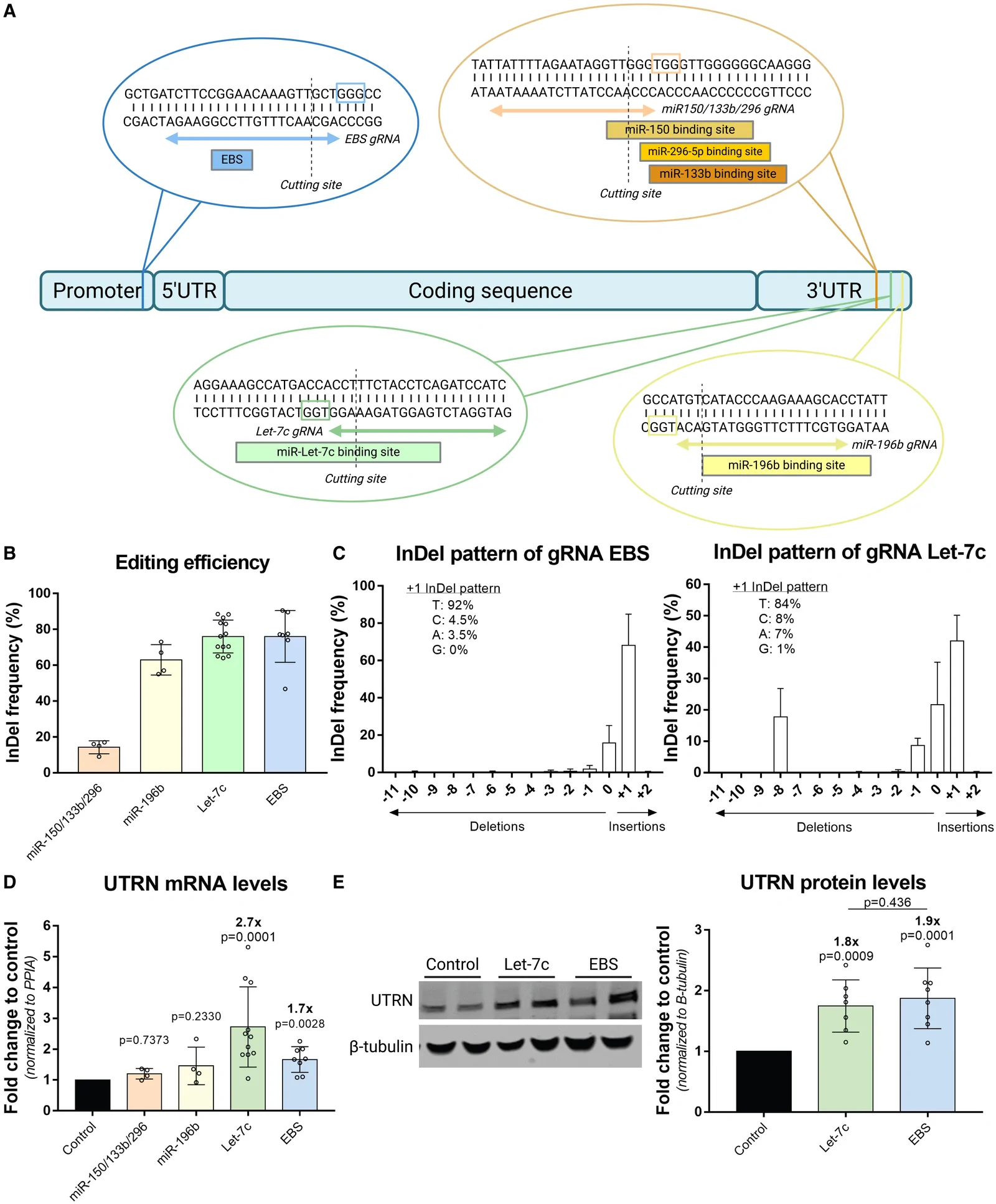
CRISPR-Cas9-mediated disruption of Let-7c BS and EBS upregulates UTRN in human DMD myoblasts (A) Scheme of UTRN gene with blow-up of guide RNA (gRNA) design to target ERF binding site (EBS); Let-7c binding site (BS); miR-196b BS; and the overlapping region of miR-150, miR-133b, and miR-296-5p BS. For each target, gRNA sequences are indicated by a colored line, PAM sites are framed, and *Sp*Cas9 cutting sites are indicated by a dotted line. (B) DNA editing efficiency at the indicated target loci after treatment with corresponding Cas9-gRNA complex in human DMD myoblasts bearing a deletion of exon 52 (hDMD-Δ52). InDel, insertions and deletions. *n* = 4–13 per group. (C) DNA indel pattern in edited myoblasts. “+1 InDel pattern” indicates the frequency of each inserted nucleotide. *n* = 6–13 per group. (D) UTRN mRNA expression normalized to peptidylprolyl isomerase A (PPIA) and represented as fold change to control. *n* = 4–11 per group. (E) UTRN protein expression. Left: representative western blot assay of control, Let-7c, or EBS edited myoblasts. Right: western blot quantification of UTRN, normalized to β-tubulin and represented as fold change to control. *n* = 7–8 per group. Each dot represents one biological replicate. *n* indicates the number of biological replicates per group. Bars represent the mean ± SD. Statistical analysis was performed using one-way ANOVA with Dunnett’s *post hoc* test (D) or paired one-way ANOVA with Tukey’s *post hoc* test (E).

To evaluate our de-repression approach, we designed gRNAs targeting previously described repressor BSs present in UTRN promoter—ERF BS (EBS)^51^— and 3′ UTR —miR-150/133b/296, miR-196b and miR-Let-7c BSs (Figure 1A).^52^ *Streptococcus pyogenes* Cas9 (*Sp*Cas9) and gRNA ribonucleoproteins (RNPs) were transfected in immortalized skeletal myoblasts derived from a DMD patient bearing an exon 52 deletion of the *DMD* gene (hDMD-Δ52). After 7 days, we quantified the editing rate and insertion/deletion (indel) pattern of each gRNA at the target locus (Figures 1B, 1C, and S1A). The gRNA targeting miR-150/133b/296 BS generated low indels (∼14%) and did not lead to an increase in UTRN mRNA expression (Figures 1B and 1D). Despite good cutting efficacy (∼63%), the gRNA targeting miR-196b BS minimally increased UTRN mRNA (Figures 1B and D). The other two gRNAs provided high editing levels and increased UTRN expression, both at the mRNA and protein levels (Figures 1D and 1E). Specifically, an insertion of one nucleotide, a thymine, was sufficient to disrupt EBS and activate transcription (Figures 1C–1E), while the Let-7c BS-targeting gRNA generated three main indels: an insertion of one nucleotide—mostly thymine—and a deletion of one or eight nucleotides (Figure 1C).

Overall, these data demonstrate that CRISPR-Cas9-mediated EBS and Let-7c BS disruption results in ∼2- to 3-fold UTRN upregulation in human DMD myoblasts. Let-7c BS editing was prioritized for further studies due to previous supporting evidence.^52^^,^^53^^,^^54^^,^^62^

### The Let-7c BS-targeting gRNA efficiently disrupts its target without major off-target effects

**Figure 2:**
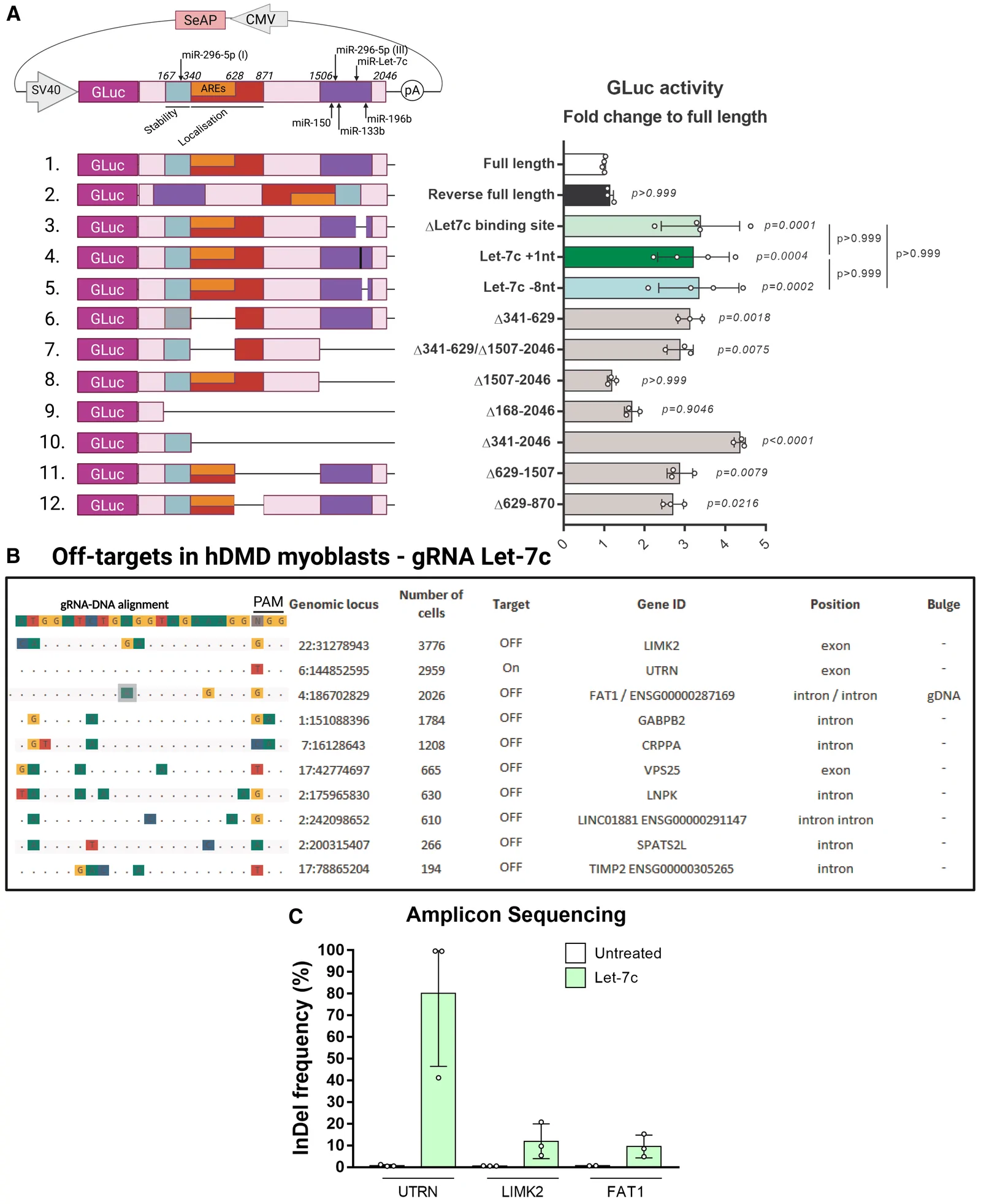
Let-7c gRNA efficiently disrupts its target without major off-target effects (A) Left: schematic representation of the dual reporter plasmid. Gaussia luciferase (GLuc) is under the control of the strong ubiquitous SV40 promoter and upstream of the 3′ UTR sequence of UTRN. The secreted embryonic alkaline phosphatase (SEAP) gene is included in the plasmid to normalize for transfection efficiency. From this full-length 3′ UTR construct, 11 additional sequences were generated, containing a full deletion of the Let-7c BS (ΔLet-7c BS), an insertion of one nucleotide (Let-7c +1 nt), a deletion of eight nucleotides (Let-7c -8 nt), or missing AREs and miR clusters. Deleted regions are indicated as Δstarting-ending nucleotide. Right: GLuc expression normalized to SEAP and represented as fold change to the full-length condition 48 h post transfection of the reporter systems. *n* = 3–4 per group. (B) Off-target analysis by Guide-seq in hDMD-Δ52 myoblasts treated with gRNA Let-7c. The top 10 genomic sequences identified are listed. On the left, alignments of the gRNA to the genomic DNA sequences are shown. Perfect matches are represented by black dots and mismatches are represented by colored boxes. Gray boxes indicate bulges between the DNA and the gRNA sequence. On the right, information about each hit is indicated. Data represent the sum of two independent experiments. (C) Amplicon-seq data from hDMD-Δ52 myoblasts treated with the Let-7c gRNA. The on-target and the first two off-target sites identified by Guide-seq were amplified and sequenced by NGS. *n* = 3 per group. Each dot represents one biological replicate. *n* indicates the number of biological replicates per group. Bars represent the mean ± SD. Statistical analysis was performed using one-way ANOVA with Tukey’s *post hoc* test.

We next evaluated the impact of the Let-7c BS-targeting gRNA editing pattern on UTRN upregulation. We generated four plasmid constructs containing the Gaussia luciferase (GLuc) reporter gene upstream of human UTRN 3′ UTR variants. One construct contained the full-length 3′ UTR sequence (variant 1), one had the whole Let-7c BS removed (variant 3), and the other two replicated the +1 or −8 indels (variants 4 and 5) observed after Let-7c BS editing (Figure 2A). In addition, these constructs also contained the secreted embryonic alkaline phosphatase (SEAP) to normalize for transfection efficiency. We transfected these plasmids into hDMD-Δ52 myoblasts and quantified GLuc and SEAP activities after 48 h. Deletion of Let-7c BS increased GLuc activity by 3.5-fold, further highlighting the crucial role of miR-Let-7c in the regulation of UTRN mRNA. Interestingly, the constructs containing the +1 and −8 indel patterns exhibited similar GLuc activity compared to the one with the full BS deletion (Figure 2A), suggesting that small indels are sufficient to cause a complete disruption of the BS.

We then generated seven other constructs to elucidate the role of 3′ UTR in human UTRN mRNA stability and translation. Based on existing literature on murine UTRN,^52^^,^^66^^,^^67^^,^^68^^,^^69^ we predicted several regulatory sequences and designed variants with deletions of AU-rich elements (AREs), miR BS clusters, and/or regions involved in stability and localization. Most variants showed an increase in activity over the full-length 3′ UTR. Variant 6 exhibited ∼3-fold upregulation of GLuc signal, confirming the role of predicted AREs in decreasing UTRN mRNA stability.^67^^,^^68^ Interestingly, variant 7, which corresponds to variant 6 without the miR BS cluster, did not show further upregulation. This observation was further confirmed with variant 8, where removal of the miR BS cluster from the full-length 3′ UTR resulted in no GLuc increase. This could suggest the presence of both repressing and stabilizing elements in the 3′ UTR terminal region of the *UTRN* gene.

Variants 9 and 10 gave the lowest and highest GLuc activity, respectively, although they differed only for the presence of a 172 base pair (bp) sequence, the 168–340 region, in variant 10. This indicates that this region is crucial for mRNA stability. Interestingly, the variants for Let-7c BS (variants 3, 4, 5) upregulated GLuc activity only slightly less than variant 10, although the latter contains a 1,700-bp deletion, again highlighting the importance of miR-Let-7c in UTRN regulation.

We then evaluated the specificity of Let-7c BS gRNA by performing a genome-wide, unbiased detection of off-target loci (Guide-seq).^70^^,^^71^ We did the analysis both in HEK-293T (Figure S1B) and hDMD-Δ52 myoblasts (Figure 2B). Although ranked differently, most of the detected off-targets were found in both cell lines, confirming that off-targets are mostly determined by the simple gRNA sequence. Within the top 10 cutting sites, two were in gene exons: (1) LIMK2 off-target is located in the 3′ UTR, not altering its coding sequence; (2) VPS25 gene is expressed at low levels in muscles. We also confirmed editing of the top 2 off-targets by performing Amplicon sequencing (Amplicon-seq) of myoblasts transfected with Let-7c gRNA (Figure 2C). Although editing was clearly observed, it remained well below the on-target. Further analyses will be necessary before this strategy can be translated into clinical application.

In summary, these data demonstrate that our CRISPR-Cas9 strategy targets UTRN 3′ UTR with high efficacy and without major off-targets.

### Disruption of Let-7c BS results in UTRN upregulation and functional improvements of human 3D engineered DMD skeletal muscles

To evaluate whether Let-7c BS editing could affect the myogenic differentiation program, we differentiated edited and unedited myoblasts into myotubes (Figure S2A). Myogenin expression, a marker of late myotube formation, was increased in myotubes compared to myoblasts, both in edited and unedited cells (Figure S2B). UTRN expression, which is known to increase during differentiation,^72^^,^^73^ was upregulated in myotubes compared to myoblasts. Importantly, UTRN mRNA levels were 2-fold higher in Let-7c BS edited myotubes compared to control myotubes, indicating that the effect of the treatment persisted after differentiation (Figure S2C). Finally, we confirmed that editing efficiency and pattern were similar in myoblasts and myotubes, indicating there was no negative selection of edited cells (Figures S2D and S2E).

**Figure 3:**
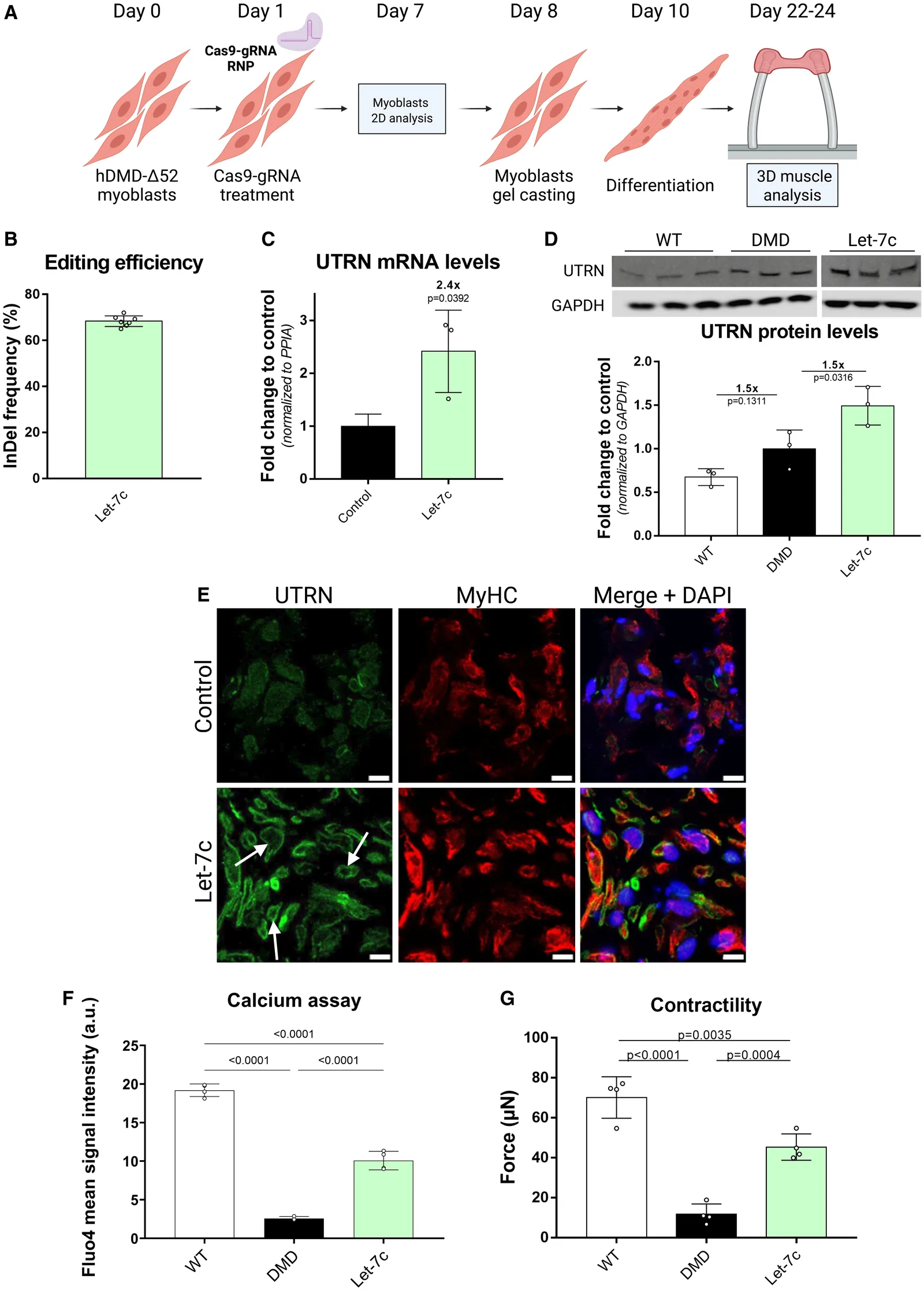
Let-7c BS disruption upregulates UTRN in human 3D engineered muscles and ameliorates calcium dysregulation and contraction (A) Workflow of 3D muscle generation from edited immortalized hDMD-Δ52 myoblasts. (B) DNA editing efficiency at the Let-7c BS in 3D muscles made from edited myoblasts. *n* = 7 per group. (C) UTRN mRNA expression normalized to PPIA and represented as fold change to unedited 3D muscles. *n* = 3 per group. (D) UTRN protein expression. Top: western blot assay of WT, DMD, or DMD-Let-7c BS edited 3D muscles. Bottom: western blot quantification of UTRN, normalized to GAPDH and represented as fold change to control. *n* = 3 per group. (E) Representative immunofluorescence staining of UTRN and myosin heavy chain (MyHC) on control DMD or DMD-Let-7c BS edited 3D muscle transverse sections. White arrows indicate UTRN signal concentration at the proximity of the sarcolemma. Scale bar: 50 μm. (F) Calcium transients assay performed on 3D muscles. Each dot represents the mean amplitude of peak signals obtained upon stimulation. *n* = 4 per group. (G) Mean contraction force measured in micro-Newtons (μN) during 12-V twitch stimulation. *n* = 4 per group. Each dot represents one 3D muscle. *n* indicates the number of whole, individual 3D muscles per group. Bars represent the mean ± SD. Statistical analysis was performed using unpaired *t* test (C) or one-way ANOVA with Dunnett’s (D) or Tukey’s (F and G) *post hoc* tests.

To evaluate our strategy in a more physiologically relevant, organoid-like setting, we harnessed our expertise in advanced *in vitro* disease and therapy modeling for muscular dystrophy^74^^,^^75^^,^^76^^,^^77^^,^^78^ to generate 3D engineered skeletal muscles from Let-7c BS edited or unedited myoblasts. Briefly, human immortalized myoblasts were embedded into a fibrin hydrogel and grown for 2 days before initiating differentiation (Figures 3A and S3A).^74^^,^^77^ After 14 days of differentiation, 3D muscles exhibited the same indel frequency and pattern as 2D cultures (Figures 3B and S3B), again indicating no negative effect of editing on the myogenic program. In addition, control and edited 3D muscles exhibited similar expression levels of several differentiation markers (Figures S3C–S3F) as well as similar differentiation and fusion indexes (Figures S3G and S3H). UTRN levels were increased in treated muscles compared to untreated ones, to levels similar to our 2D experiments (Figures 3C, 3D, and S3I). Immunofluorescence staining of cross-sections of edited 3D muscles showed that UTRN was upregulated and concentrated at the border of each fiber (Figure 3E). This confirms that UTRN upregulation remains stable after differentiation and suggests sarcolemmal localization, which might provide protection from contraction-induced damage.

In DMD, loss of dystrophin enhances sarcolemma susceptibility to contraction-induced damage via increased calcium influx into dystrophic fibers, leading to downstream protease activation, metabolic dysfunction, loss of muscle function, and necrosis. To assess whether UTRN can correct DMD-associated sarcolemmal fragility and impaired calcium homeostasis, we measured intracellular calcium transients. Wild-type (WT), DMD, or DMD-Let-7c BS edited 3D muscles were exposed to the Fluo-4 fluorescent calcium indicator and subjected to 10 V electrical stimulation. In DMD 3D muscles, calcium release was significantly reduced compared to WT muscles, recapitulating calcium defects observed in the pathology. Notably, calcium transients improved significantly in DMD-Let-7c BS edited 3D muscles (Figures 3F and S3J). We then evaluated the effect of the treatment on 3D muscle contraction (Figures S3K and S3L). As expected, DMD muscles generated significantly less force during twitch contraction than WT muscles. Interestingly, Let-7c BS edited muscles showed a significant increase in force generation compared to untreated DMD muscles (Figure 3G).

Overall, these data demonstrate that upregulation of UTRN through miR-Let-7c BS disruption provides functional benefits by improving calcium signaling and contractility defects in 3D human DMD muscles.

### Evaluation of gRNAs targeting Let-7c BS for murine UTRN upregulation

**Figure 4:**
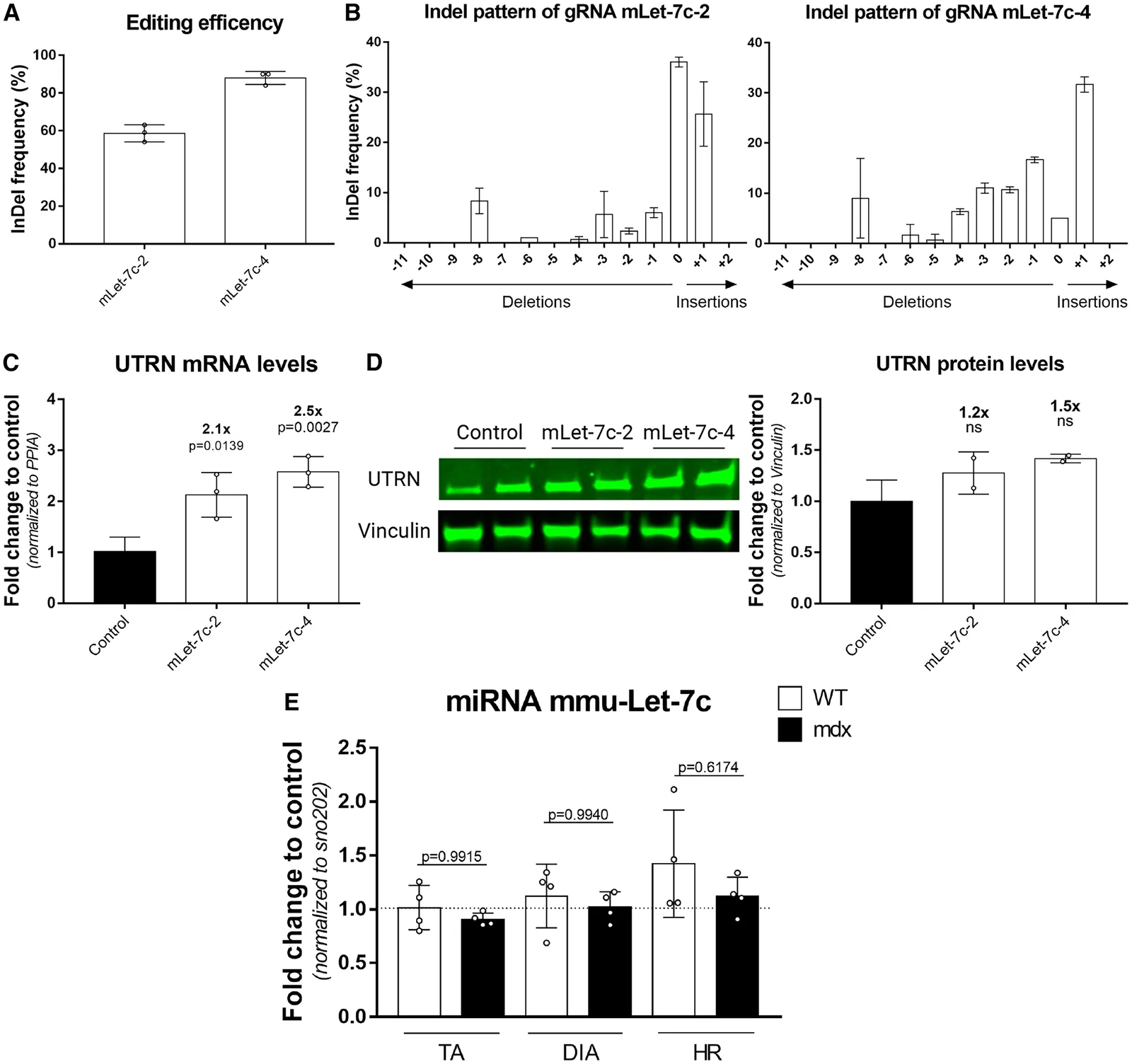
miR-Let-7c BS disruption induces a 2-fold increase in UTRN expression in C2C12 mouse myoblasts (A) DNA editing efficiency at the Let-7c BS after treatment with corresponding Cas9-gRNA complex in murine C2C12 myoblasts. *n* = 3 per group. (B) DNA indel pattern in gRNA mLet-7c-2 (left) and gRNA mLet-7c-4 (right) edited myoblasts. *n* = 3 per group. (C) UTRN mRNA expression normalized to PPIA and represented as fold change to control. *n* = 3 per group. (D) UTRN protein expression. Left: western blot assay of mLet-7c-2 or mLet-7c-4 treated myoblasts. Right: western blot quantification of UTRN, normalized to vinculin and represented as fold change to control. *n* = 2 per group. ns, non-significant. (E) Murine Let-7c miRNA (mmu-Let-7c) expression was measured by qPCR in the tibialis anterior (TA), diaphragm (DIA), and heart (HR) of WT and *mdx* mice, normalized to sno202 expression, and represented as fold change to the expression in the TA of WT mice. *n* = 4 mice per group. Each dot represents one biological replicate. *n* indicates the number of biological replicates per group. Bars represent the mean ± SD. Statistical analysis was performed using one-way ANOVA with Dunnett’s (C and D) or Tukey’s (E) *post hoc* tests.

To evaluate our Let-7c BS disruption strategy in the *mdx* mouse model, we first checked interspecies sequence conservation and found that miR-Let-7c sequence is fully conserved between mice and humans, while there is a single nucleotide mismatch between the two Let-7c BS (Figure S4A). We thus designed two gRNAs targeting the murine Let-7c BS, mLet-7c-2, and mLet-7c-4 (Table S1 and Figure S4B). Both gRNAs demonstrated good editing efficiency in murine C2C12 myoblasts (Figure 4A), with indel patterns similar to the human gRNA (Figure 4B). In addition, both gRNAs induced detectable upregulation of UTRN at the mRNA and protein levels (Figures 4C and 4D), confirming that Let-7c-mediated UTRN post-transcriptional downregulation is conserved in mice. Since gRNA mLet-7c-4 disrupted the sequence interacting with the miR seed, exhibited better on-target efficiency, and induced a higher UTRN upregulation compared to gRNA mLet-7c-2, we chose this gRNA for further studies. Finally, since some miRs are known to be dysregulated in DMD muscle tissues,^79^^,^^80^^,^^81^ we performed a qPCR analysis to confirm that both WT and *mdx* mice express similar levels of mmu-Let-7c in cardiac, respiratory, and skeletal muscles (Figure 4E).

In summary, we defined and validated two efficient gRNAs disrupting Let-7c BS in murine myoblasts and selected gRNA mLet-7c-4 for subsequent *in vivo* analyses.

### Intramuscular injection of CRISPR AAV9 upregulates UTRN and ameliorates functional parameters in the *mdx* mouse model

**Figure 5:**
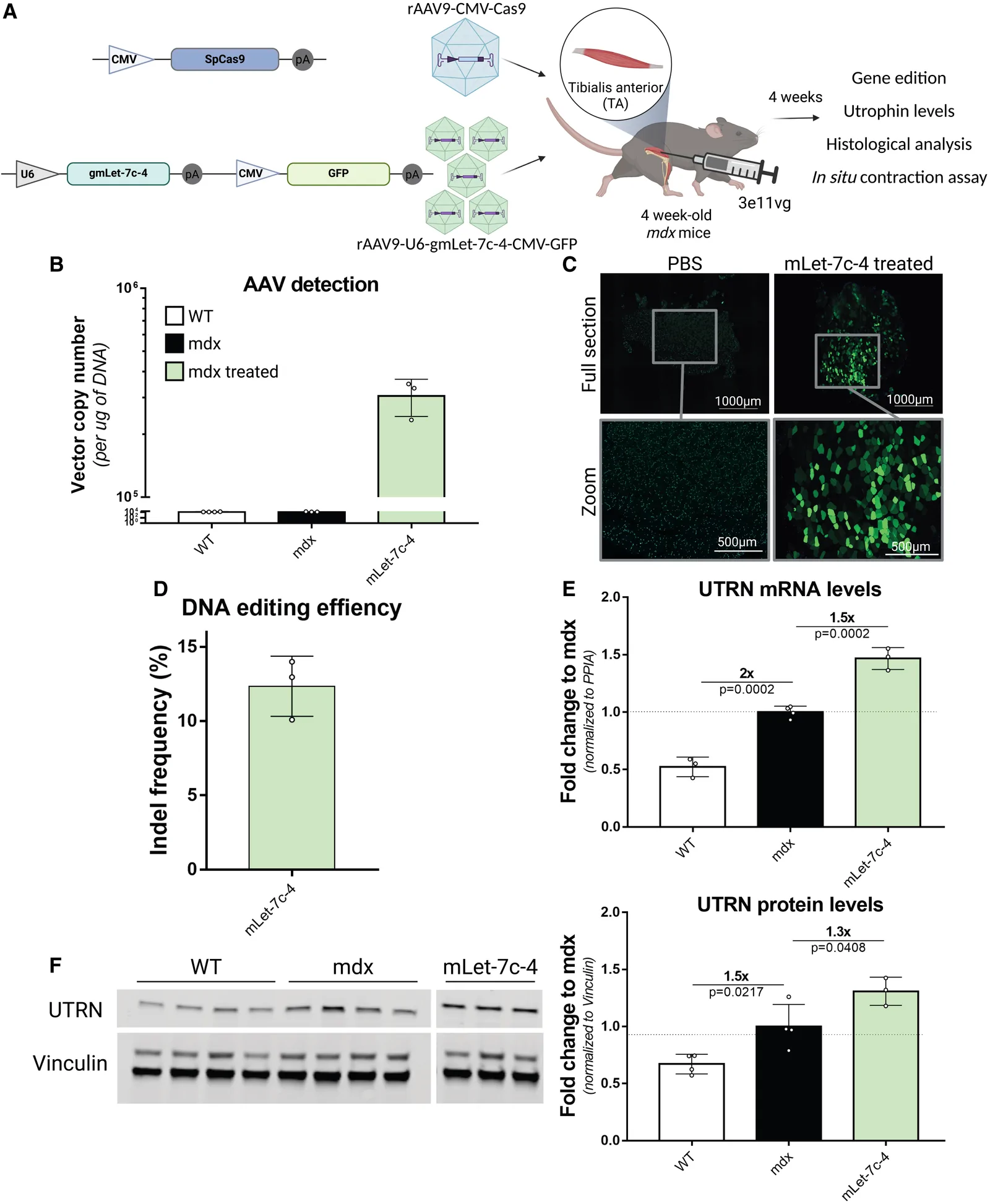
Intramuscular AAV9 delivery of Cas9 and Let-7c BS gRNA results in upregulation of UTRN in *mdx* mice (A) Workflow of the experiment. 4-week-old *mdx* mice were injected with a total of 3e11 vector genomes (vg) of AAV9-Cas9 and AAV9-gRNA-mLet-7c-4 intramuscularly in one tibialis anterior (TA) with a 1:5 Cas9:gRNA ratio. WT and *mdx* groups treated with PBS were used as controls. After 4 weeks of treatment, TAs were collected for analysis. (B) Quantification of total AAV9 vector copy number in the TA of PBS or gRNA-mLet-7c-4-treated mice. (C) Native GFP signal in TA transverse sections acquired with the AxioScan device. Zoom: 10×. Scale bars are indicated. (D) DNA editing efficiency at the Let-7c BS measured by NGS in gRNA-mLet-7c-4-treated mice. (E) UTRN mRNA expression normalized to PPIA and represented as fold change to *mdx* group. (F) UTRN protein expression. Left: western blot assay of PBS or gRNA-mLet-7c-4-treated mdx mice TAs. Right: western blot quantification of UTRN, normalized to vinculin and represented as fold change to mdx. Each dot represents one TA. *n* = 3–4 TAs per group. Bars represent the mean ± SD. Statistical analysis was performed using one-way ANOVA with Dunnett’s *post hoc* test.

To evaluate our approach into *mdx* mice, we generated two recombinant AAV9: the first one encoding for the *Sp*Cas9 sequence under the control of the cytomegalovirus (CMV) promoter, and the second one for the mLet-7c-4 gRNA under the control of the human U6 promoter and GFP under the CMV promoter, to follow vector biodistribution (Figure 5A). A total of 3e11 vector genomes (vg) was administered to 4-week-old *mdx* mice via intramuscular (i.m.) injection^82^^,^^83^^,^^84^ in the tibialis anterior (TA) in a 1:5 ratio, with five times more AAV gRNA than AAV Cas9. WT and *mdx* mice were injected with PBS as controls. 4 weeks after treatment, TAs were collected and analyzed. Vector copy-number analysis and GFP signal revealed efficient muscle transduction and good transgene expression, respectively (Figures 5B and 5C). DNA sequencing of the Let-7c BS by next-generation sequencing revealed ∼12% of editing (Figure 5D). As expected, UTRN mRNA and protein levels were 2-fold higher in *mdx* mice compared to WT, due to the natural endogenous compensatory mechanism. Interestingly, an additional increase in UTRN mRNA (∼1.5-fold) and protein (∼1.3-fold) was observed after editing (Figures 5E, 5F, and S5B).

**Figure 6:**
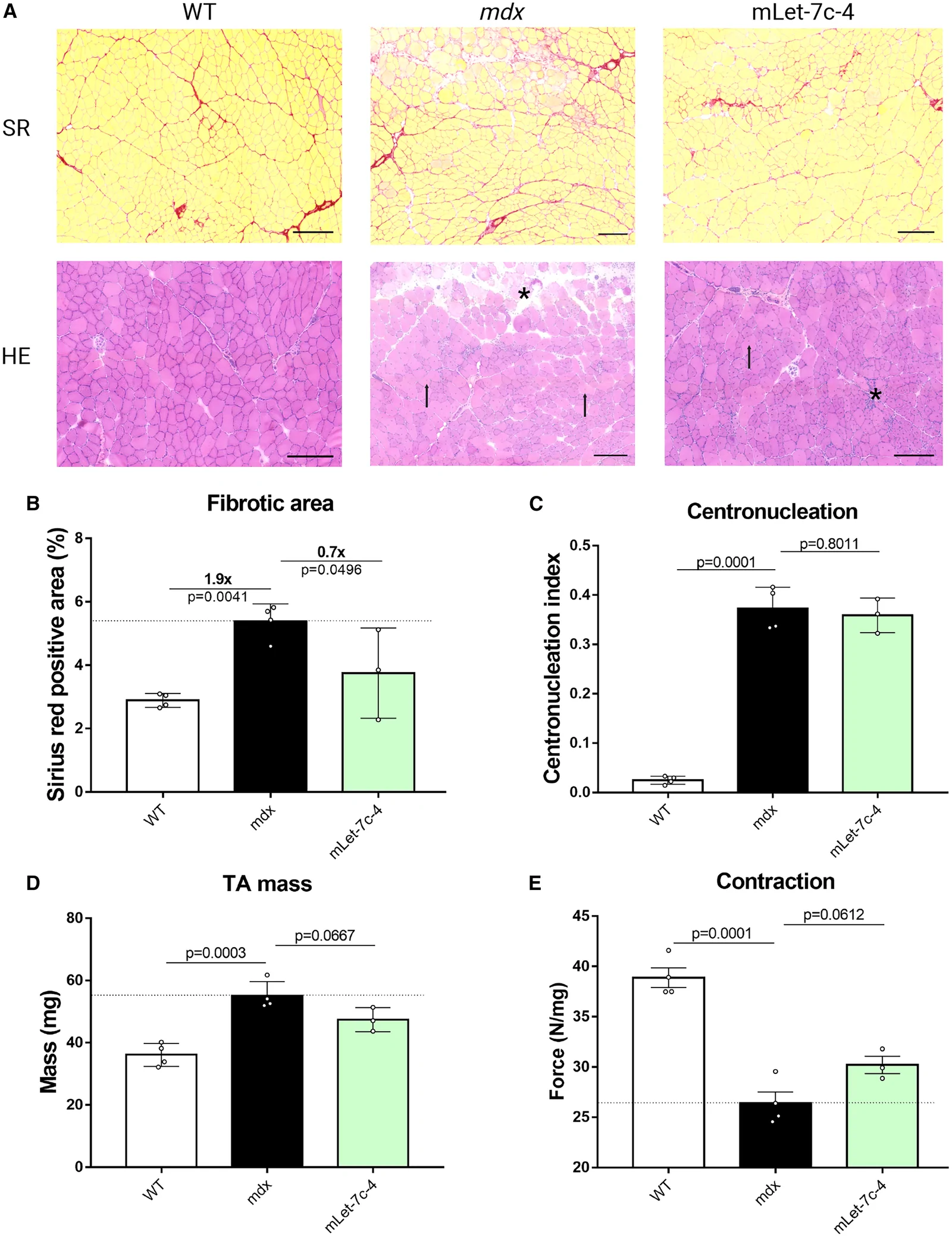
Intramuscular AAV9 delivery of Cas9 and Let-7c BS gRNA tends to ameliorate DMD histological and functional parameters in *mdx* mice (A) Representative images of Sirius red (SR; top) and hematoxylin and eosin (HE; bottom) stainings of TA transverse sections. Regions of necrosis and infiltration are indicated by black stars. Examples of centrally nucleated fibers are indicated by a black arrow. Scale bars: 200 μm. (B) Quantification of SR-positive area (collagen deposition). (C) Quantification of centrally nucleated fibers. (D) TA mass measured in milligrams (mg). (E) Tetanic force measured in Newtons (N) during TA *in situ* contraction assay and normalized by TA weight (N/mg). Each dot represents one TA. *n* = 3–4 TAs per group. Bars represent the mean ± SD. Statistical analysis was performed using one-way ANOVA with Dunnett’s *post hoc* test.

We then performed histological analysis to evaluate the therapeutic benefits of the treatment. Fibrosis was more pronounced in *mdx* mice compared to WT controls, and it was reduced by 30% upon editing (Figures 6A and 6B). Hematoxylin and eosin staining showed that treated muscles exhibited overall improved organization, although some areas still showed immune cell infiltration (Figure 6A). Central nucleation, a marker of regenerating fibers, remained unchanged (Figure 6C). The TA mass, which is increased in *mdx* mice compared to WT due to fibro-fatty deposition, was reduced after treatment, although the change was not statistically significant (Figure 6D).

We then performed an *in situ* TA tetanic contraction assay. As expected, *mdx* specific force was weaker compared to WT, reflecting lower resistance to fatigue, but showed a trend toward improvement upon treatment (Figure 6E).

Overall, our data indicate that AAV9 i.m. delivery enables *in vivo* editing in *mdx* mice, induces upregulation of UTRN, and ameliorates several histological and functional hallmarks of DMD.

### Systemic injection of CRISPR AAV9 upregulates UTRN in the *mdx* mouse model

**Figure 7:**
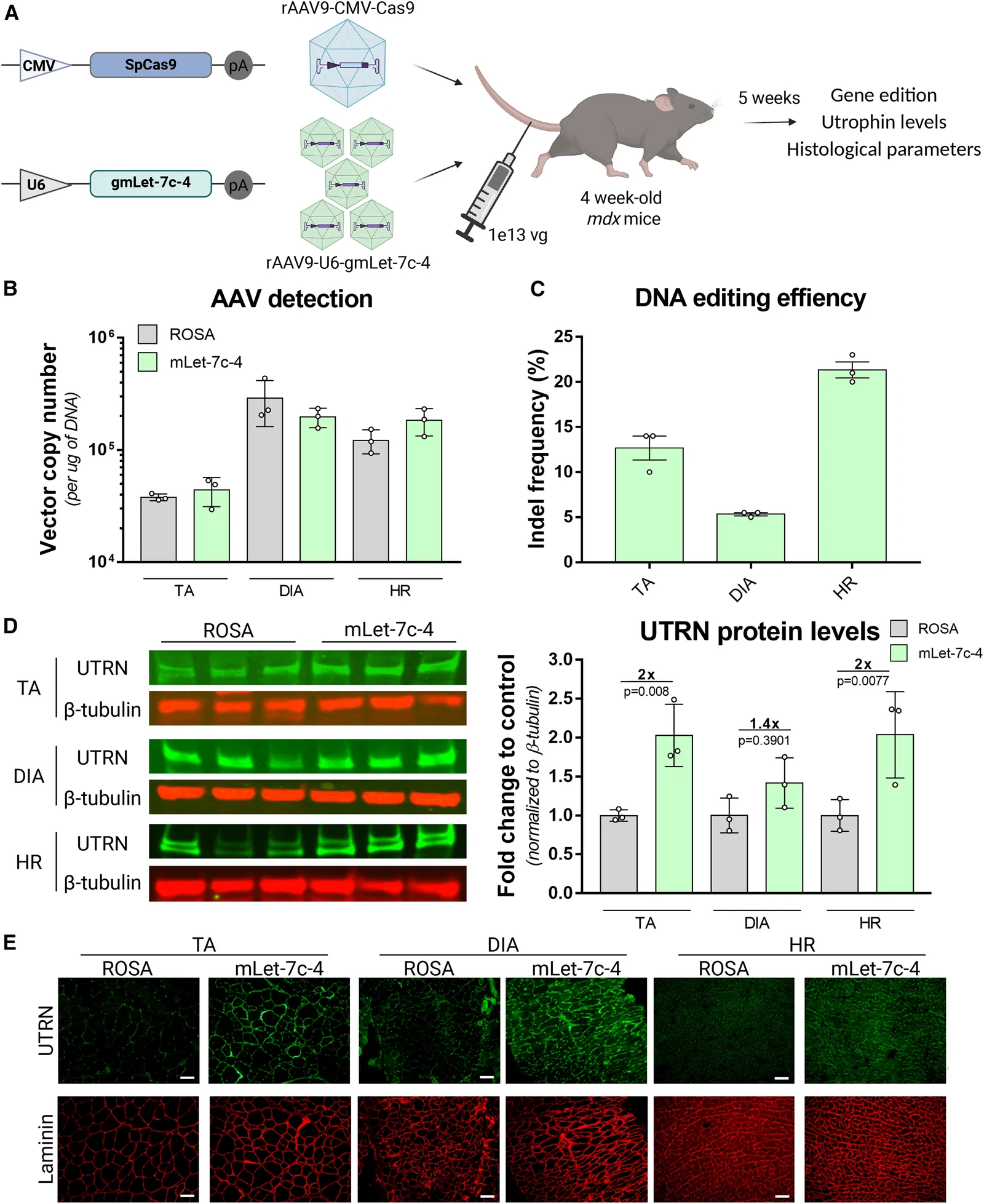
Systemic AAV9 delivery of Cas9 and Let-7c BS gRNA results in upregulation of UTRN in *mdx* mice (A) Workflow of the experiment. 4-week-old *mdx* mice were injected with a total of 1e13 vg of AAV9-Cas9 and AAV9-gRNA-mLet-7c-4 in the tail vein with a 1:5 Cas9:gRNA ratio. A group treated with a ROSA-targeting gRNA was used as control. After 5 weeks of treatment, tibialis anterior (TA), diaphragm (DIA), and heart (HR) were collected for analysis. (B) Quantification of total AAV9 vector copy number in the TA, DIA, and HR of gRNA-ROSA or gRNA-mLet-7c-4-treated mice. (C) DNA editing efficiency at the Let-7c BS measured by NGS in the TA, DIA, and HR of gRNA-mLet-7c-4-treated mice. (D) UTRN protein expression. Left: western blot assay of gRNA-ROSA or gRNA-mLet-7c-4-treated *mdx* mice TA, DIA, and HR. Right: western blot quantification of UTRN, normalized to β-tubulin and represented as fold change to ROSA-treated mice. (E) Immunofluorescence staining of UTRN in TA, DIA, and HR of control or treated mice indicates sarcolemma localization of the upregulated protein. Scale bars: 100µm. Each dot represents one mouse. *n* = 3 mice per group. Bars represent the mean ± SD. Statistical analysis was performed using one-way ANOVA with Sidak’s *post hoc* test.

To evaluate the systemic effect of our treatment, we performed tail-vein injection of 1e13 vg per mouse of the aforementioned AAV9-Cas9 and AAV9-gRNA-mLet-7c-4 (1.7e12 vg and 8.3e12 vg, respectively) in 4-week-old *mdx* mice. A control group was injected with an AAV encoding for a gRNA targeting the non-essential *Rosa26* locus (Table S1).^59^ 5 weeks post injection, relevant organs—TA, diaphragm (DIA) and heart (HR)—were collected for analysis (Figure 7A). We confirmed muscle transduction by measuring vg copy number (Figure 7B) and Let-7c BS editing in the HR (∼21%), TA (∼13%), and DIA (∼5%) (Figures 7C and S5A). Western blot analysis showed an increase of UTRN levels of ∼2-, ∼2-, and ∼1.4-fold in the HR, TA, and DIA of mLet-7c-4-treated mice, respectively (Figure 7D). Interestingly, immunofluorescence staining confirmed correct UTRN localization at the sarcolemma (Figure 7E). Although fibrosis was decreased in the DIA (Figure S6A), no other significant differences were observed at the histological level (Figures S6B–S6D), probably due to the relatively low editing efficiency.

In conclusion, we demonstrated that our CRISPR-Cas9 strategy has the potential to upregulate endogenous UTRN expression *in vivo* in *mdx* muscles.

## Discussion

Upregulating UTRN is an attractive therapeutic approach for DMD as it could be applied to all DMD patients regardless of their genetic defect, it is potentially less immunogenic than dystrophin-based approaches, and it may help ameliorate DMD-associated cardiomyopathy.^43^^,^^64^ Our study describes the *in vitro* and *in vivo* potential of a CRISPR-based approach to de-repress endogenous full-length UTRN by disrupting its BS for the repressor miR-Let-7c in human and murine cells, muscle organoids, and the *mdx* mouse model. While miR-Let-7c BS-targeting strategies have already been described, they relied on masking the entire BS using antisense oligonucleotides, required repeated administration and showed limited efficacy.^53^^,^^54^ More recently, UTRN transcriptional activation has also been evaluated^63^^,^^64^^,^^65^ and, although interesting, it requires continuous expression of the effector protein. Other groups described a dual-gRNA editing strategy to permanently remove a cluster of miR BS within the *UTRN* 3′ UTR sequence, reporting a 1.5- to 2-fold UTRN upregulation in DMD immortalized myoblasts and DMD human induced pluripotent stem cells.^62^^,^^85^ Here, using reporter plasmids in human DMD myoblasts, we further demonstrated that this miR BS cluster region might contain elements important for mRNA stability or translation and that miR-Let-7c is the main driver of UTRN post-transcriptional repression. In addition, the disruption of Let-7c BS alone is sufficient to achieve UTRN de-repression both *in vitro* and *in vivo* with a single administration, offering the potential for a one-time treatment. Although partially beneficial, UTRN upregulation remained modest, in line with microRNA biology, as microRNAs orchestrate complex regulatory programs by fine-tuning the expression of hundreds of target genes within a narrow range (20%–40%) rather than acting as on/off regulators.^86^^,^^87^ Consequently, disrupting a single miR BS cannot fully de-repress UTRN, suggesting that combinatorial strategies targeting multiple sites or pathways may be required for a more robust effect. Our strategy has the additional advantage of requiring a single gRNA, which limits potential genotoxicity and facilitates delivery. Although our gRNA showed good specificity and low genotoxic risk, the generation of DNA double-strand breaks can potentially cause unintended genomic rearrangements such as translocations or chromothripsis.^88^^,^^89^^,^^90^^,^^91^ Thanks to the observation that even a single nucleotide change is sufficient to disrupt the Let-7c BS as efficiently as a full BS deletion, we can envision the use of safer editors such as base-editing enzymes.^92^^,^^93^ In addition, base editing will allow targeting multiple repressor BS (e.g., EBS and Let-7c BS) to achieve additive or synergistic UTRN de-repression at transcriptional and post-transcriptional levels.

Although editing efficiency in the TA muscle is comparable between AAV intramuscular and systemic injections, the distribution of edited fibers is likely more heterogeneous after local injection, which could explain the differences in UTRN upregulation. While our approach upregulated UTRN expression and partially ameliorated the dystrophic phenotype *in vivo*, editing efficiency remained low and further optimizations will be necessary before clinical translation. Using myotropic AAV capsids such as AAVMYO,^94^ MyoAAV,^95^ or LICA1^96^ could enhance muscle-targeted delivery and thus increase editing efficiency while limiting liver targeting and associated toxicity. The large 4.1-kb size of *Sp*Cas9 requires a dual-AAV strategy, which increases vector doses and the risk of AAV capsid-directed immune response. Conversely, an all-in-one AAV approach would decrease total AAV dose and ensure co-delivery of Cas9 and gRNA. However, the limited packaging size of AAVs would necessitate the use of smaller nucleases^97^^,^^98^ such as *Sa*Cas9,^99^ Cas12f,^100^ NanoCas,^101^ or CoCas.^102^ Alternatively, lipid nanoparticles (LNPs) could be used as a transient, immunologically safe, and re-dosable delivery vector to circumvent the size constraints associated with AAVs and reduce the lifespan of the editing tool, thus lowering genotoxicity.^103^

While several UTRN-based therapies have been extensively studied in animal models, only pharmacological upregulation has been evaluated in human clinical studies, where it failed to provide benefits to the patients.^104^ Therefore, it is key to have predictive models capable of assessing the performance of UTRN-based strategies in a human, pre-clinical setting. Most studies using human cells have shown UTRN upregulation in conventional 2D models, which have limited capacity to assess functional benefits.^62^^,^^72^^,^^105^^,^^106^^,^^107^^,^^108^ To overcome these limitations we harnessed *in vitro* 3D modeling to evaluate gene-editing-mediated UTRN upregulation, demonstrating improvements in calcium regulation and contractile properties, as previously observed using pharmacological UTRN upregulators.^109^ WT and DMD myoblasts used in our study were derived from different individuals, hence limiting some comparative analysis: future studies using isogenic cell pairs will further complement our results.

Finally, recent studies demonstrated that UTRN can colocalize with dystrophin or micro-dystrophin at the same muscle membrane,^110^^,^^111^ suggesting that our UTRN upregulation strategy could be combined with strategies restoring dystrophin expression.

In summary, we provide *in vitro* and *in vivo* evidence that targeted disruption of miR-Let-7c BS can upregulate UTRN expression, offering a therapeutic gene-editing approach amenable to all DMD pathogenic variants. While full-length dystrophin recovery is widely considered the optimal therapeutic approach, the existing limitations related to its delivery and patient immune response highlight the potential of our UTRN de-repression strategy as a viable alternative in the treatment of DMD.

## Materials and methods

### Ethical statement

All animal procedures were performed following the European guidelines for the human care and use of experimental animals. Animal experimentations were approved by the Ethical Committee for Animal Experimentation C2AE-51 of Evry under number APAFIS#29497-2020102611378971 v2 and DAP 2020-001-B. All C57BL/10ScSn-Dmd*mdx*/J (BL10/*mdx*) mice were bred in the Experimental Functional Research Exploration Center (CERFE) facility, Genopole. Work with human cells in Dr. Tedesco’s laboratory was performed under approval of the NHS Health Research Authority Research Ethics Committee reference no. 13/LO/1826; IRAS project ID no. 141100.

### Cell cultures

Immortalized human WT (AB1190) or DMD (KM1328) myoblasts were obtained from the Myoline Plateform (Institut de Myologie, France) and maintained in Skeletal Muscle Cell Growth Medium (C-23060, PromoCell, Germany) supplemented with 20% fetal bovine serum gold (PAA) and 1% penicillin-streptomycin (Invitrogen) at 37°C with 5% CO_2_. Cell differentiation was performed using the PromoCell Skeletal Muscle Differentiation Medium (C-23061, PromoCell, Germany). Immortalized WT murine C2C12 and HEK-293T cells were maintained in DMEM (Invitrogen) supplemented with 10% fetal bovine serum gold (PAA) and 1% penicillin-streptomycin (Invitrogen) at 37°C with 5% CO_2_.

### AAV plasmid DNA cloning and AAV production

The AAV plasmid DNA (pDNA) encoding *Sp*Cas9 under the control of the CMV promoter (pX551-CMV-*Sp*Cas9) is from the Hewitt lab (Addgene plasmid #107024). To generate the AAV pDNA encoding the gRNA (U6 promoter) and GFP (CMV promoter), we used the pAAV-U6-sgRNA-CMV-GFP plasmid (Hetian Lei lab, Addgene plasmid #85451), replaced the original gRNA scaffold with an optimized version,^112^ and cloned the desired gRNA protospacer sequence via SapI digestion.^113^ All constructs were validated by digestion and Sanger sequencing. Recombinant single-stranded AAV9 was then produced using a triple-transfection protocol in HEK293 cells in suspension. AAVs were purified by affinity chromatography (AVB Sepharose; GE Healthcare, Chicago, IL), formulated in sterile phosphate-buffered saline (PBS) containing 0.001% of pluronic acid (F68; Sigma-Aldrich, Saint Louis, MO), and stored at −80°C. Production titer was determined by real-time quantitative polymerase chain reaction (qPCR) using primers and probes located in the CMV promoter (forward: 5′-CATCAATGGGCGTGGATAGC-3′; reverse: 5′-GGAGTTGTTACGACATTTTGG-3′).

### GLuc reporter plasmids

*UTRN* 3′ UTR sequence was recovered from Ensembl: ENST00000367545.8. All 3′ UTR reporter constructs were generated by GenScript Biotech (Leiden, the Netherlands). The 3′ UTR of *UTRN* was inserted in the pEZX-GA02 reporter cloning vector (ZX-104, Genecopoeia), downstream of the GLuc. This vector also contains SEAP. Plasmids were controlled by enzymatic digestion and Sanger sequencing. They were transformed into XL-10 bacteria for amplification and extracted using the NucleoSpin Plasmid kit (740588.50, Macherey-Nagel) following manufacturer recommendations.

For transfection, 1e4 hDMD-Δ52 myoblasts were seeded in a 96-well plate. The next day, the plasmids were transfected in cells using Lipofectamine 3000 (L3000008, Thermo Fisher) according to manufacturer’s instructions. Briefly, 100 ng of the pEZX-GA02-3′ UTR variant and 0.2 μL of P3000 reagent were diluted in 5 μL of OPTI-MEM. Separately, 0.3 μL of Lipofectamine 3000 was diluted in 5 μL of OPTI-MEM. Both were then mixed and incubated for 15 min at room temperature. The mixture was diluted with OPTI-MEM to a final volume of 100 μL. Forty-eight hours after transfection, supernatant was collected for enzymatic dosage.

### Enzymatic dosage

GLuc activity was measured using the following protocol: cell supernatant was diluted 1:10 in 1× DPBS (Thermo Fisher) and distributed in a white 96-well OptiPlate. We diluted coelenterazine (C3230-50UG, Sigma-Aldrich) 1:500 in DPBS before adding it to each sample. Luciferase light units were then measured using the EnSpire Multimode Plate Reader (PerkinElmer, Courtaboeuf, France). Transfection efficiency was controlled by quantification of the SEAP signal in diluted supernatant (1:20) using the Phospha-Light SEAP Reporter Gene Assay System (T1015, Thermo Fisher). GLuc values were then normalized by SEAP measurements.

### gRNA design

gRNAs targeting repressor BS were designed using the Benchling online tool (https://benchling.com). They were chosen based on their predicted efficiency, specificity and location from the target site. All gRNA sequences are presented in Table S1.

### Nucleofection

Chemically modified gRNAs were produced by Synthego (Redwood City, CA) and diluted according to manufacturer’s instructions. RNP complexes were generated by mixing 2 μL of 30 μM gRNA with 1 μL of 30 μM *Sp*Cas9 protein (kind gift from Dr. JP Concordet, Museum National d'Histoire Naturelle, Paris), 1 μL of 100 μM electroporation enhancer (1075916, Integrated DNA Technologies, Coralville, IA), and 0.3 μL of 10× HEPES buffer. The mix was incubated for 10 min at room temperature. 2e5 cells were then transfected with the RNP mix using the P5 Primary Cell 4D-Nucleofector X kit (V4XP-5032, Lonza) and the C2C12 program in the Lonza 4D Nucleofector. Cells were harvested 7 days after nucleofection.

### DNA analysis

For *in vitro* studies, 5e4 cells were collected and centrifuged for 3 min at 1,300 rpm to recover the cell pellet. Genomic DNA was extracted using the QuickExtract DNA Extraction Solution (Lucigen, Middelton, WI). DNA-containing solution was diluted 1:2 in DNAse/RNAse free H_2_O. Indel quantification was performed using 10 μL of genomic DNA solution to amplify the region that spans the cutting site of each gRNA using KAPA2G Fast ReadyMix (Kapa Biosystem, Wilmington, MA) and the primer pairs shown in Table S2. PCR amplicons were sequenced by Sanger sequencing (Genewiz, Takeley, UK), and the percentage of insertions and deletions was calculated using the ICE^114^ or TIDE^112^ software. For *in vivo* studies, genomic DNA was isolated from TA, DIA, and HR muscles from *mdx* mice using the NucleoMag Pathogen kit (744210.4, Macherey-Nagel) and the KingFisher device. After elution, DNA was quantified using the NanoDrop ND8000 spectrophotometer (Thermo Fisher Scientific).

### Quantification of vg copy number

AAV genome copy number in tissue samples was quantified using real-time TaqMan quantitative PCR analysis (ABI 7500) with primers and probe directed against the CMV promoter. Genomic DNA was mixed with 2× TaqMan Universal PCR master mix (Life Technologies, Carlsbad, CA) and 20× primer/probe mix. A plasmid containing the target sequence was used to generate a standard curve ranging from 1e1 to 1e8 copies per μL. Mouse Titin was used to normalize AAV copy number.

### Amplicon-seq

Indel quantification from mouse genomic DNA was performed by Amplicon-Seq. Amplicons were generated by two rounds amplifications. First, a target-specific amplification was performed using the Phusion High-Fidelity DNA Polymerase kit (M0530S, New England Biolabs) and the following PCR program: (1) 3 min at 98°C; (2) 32 cycles of 10 s at 98°C, 10 s at the specific primer Tm, and 15 s at 72°C; and (3) 5 min at 72°C. The amplicons were then purified using the Agencourt AMPure XP beads kit (Beckman Coulter, Brea, CA, USA) and used for the second amplification with specific barcoded primers. The final amplicons were gel purified using NucleoSpin Gel and PCR clean-up kit (740609, Macherey-Nagel) following the manufacturer’s instructions. The final products were eluted in IDTE buffer (11-01-02-05, Integrated DNA Technologies). After purification, the PCR products were pooled in equimolar concentrations and delivered to the NGS facility at Imagine Institute (Paris, France). 150-bp paired-end sequencing was performed on an Illumina NovaSeq instrument. The amplicons were cleaned, quality filtered, demultiplexed, and processed using the CRISPResso V2 online tool (https://crispresso.pinellolab.partners.org/submission).^115^

### Off-target analysis by Guide-seq

DNA library preparation for GUIDE-seq analysis was performed as previously described.^70^^,^^71^ hDMD-Δ52 myoblasts were transfected with 80 pmol of double-stranded oligodeoxynucleotide (dsODN) and a Cas9/gRNA RNP (1:2 ratio). After 5 days in culture, DNA was isolated using the QIAamp DNA mini kit following manufacturer’s instructions (QIAGEN). DNA was sonicated (Bioruptor Pico sonicator) to generate fragments of 400–900 bp. These fragments were ligated to adaptors, and two steps of DNA amplification were performed using the KAPA HyperPrep Kit (KK8504, Roche). The libraries were then purified, and concentration was measured using the qPCR KAPA libraries quantification kit (Roche) and Qubit device. Average length was estimated with the Tape Station bioanalyzer 2100 (Agilent). The libraries were pooled, diluted to a final concentration of 4 nM, and loaded onto a MiSeq flow cell. Subsequent steps included demultiplexing, consolidation of PCR duplicates, identification of on-target cleavage sites, detection of off-target activity, and data visualization, which were performed using an in-house GUIDE-seq analysis pipeline available on GitHub (GitHub - gcorre/GNT_GuideSeq: pipelines for genomic analysis).^116^

### RNA analysis

For *in vitro* studies, total RNA was extracted using the RNeasy Micro kit (Qiagen, Hilden, Germany) according to manufacturer’s instructions. For *in vivo* studies, total RNA was extracted using the Nucleozol reagent (740404200, Macherey-Nagel) according to manufacturer’s instructions. RNA was then reverse transcribed using the Transcriptor First Strand cDNA Synthesis Kit (Roche, Basel, Switzerland) according to manufacturer’s instructions. qPCR assay was performed using the Maxima Syber Green/Rox mix (Life Scientific, Thermo Fisher Scientific, Waltham, MA, US) and the following primers for UTRN amplification: forward primer 5′-ACGAATTCAGTGACATCATTAAGTCC-3′, reverse primer 5′ATCCATTTGGTAAAGGTTTTCTTCTG-3′.

UTRN expression levels were normalized to the peptidylprolyl isomerase A (PPIA) or GAPDH housekeeping genes and represented as fold change (2^−ΔΔCt^) to control, where ΔCt = Ct_(UTRN)_ – Ct_(housekeeping)_ represents the normalized UTRN Ct value, and ΔΔCt = ΔCt_(treated)_ − ΔCt_(control)_, the UTRN expression change upon treatment. A template-free condition was used as negative control.

### Protein analysis

Human cells, mouse cells, or mouse organs were homogenized in RIPA buffer (R0278, Sigma-Aldrich) supplemented with protease inhibitors (11697498001, Sigma-Aldrich). The homogenate was centrifuged for 20 min at 8,000 rpm, 4°C, and total protein in the supernatant was quantified using the Pierce BCA Protein Assay Kit (Thermo Fisher) following manufacturer’s instructions. For western blot, 30 μg of total protein was mixed with NuPAGE LDS sample buffer (NP0007, Invitrogen) and β-mercaptoethanol (11528926, Fisher Scientific) and denatured for 5 min at 100°C. Samples were then loaded on a NuPAGE 3%–8% TRIS-Acetate Gel (Novex, Life Technologies) and run in NuPAGE Tris-Acetate SDS Running Buffer (LA0041, Thermo Fisher) with NuPAGE Antioxidant (Invitrogen, NP0005) for 30 min at 50 V then 1 h 30 min at 120 V before transferring on a nitrocellulose membrane (88018, Thermo Fisher). Liquid transfer was performed in NuPAGE Tris-Acetate SDS Running Buffer for 2 h at 350 mA. The HiMark (LC5699, Thermo Fisher) or PageRuler (26619, Thermo Fisher) prestained protein ladders were used as weight markers. The membrane was then blocked for 1 h at room temperature in blocking buffer (927-70003; LI-COR; USA) and incubated overnight at 4°C with the following primary antibodies: mouse anti-UTRN (1:100, sc-33700, Santa Cruz Biotechnology), mouse anti-GAPDH (1:5,000, MAB374, Sigma-Aldrich), mouse anti-α-actinin (H-2) (1:1,000, sc-17829, Santa Cruz Biotechnology), mouse anti-vinculin (1:2,000, V9131, Sigma-Aldrich), and rabbit anti-β-tubulin (ab6046, Abcam). The next day, the membrane was incubated for 2 h with species-specific secondary antibodies. The Odyssey Imaging System (LI-COR Biosciences; USA) was used to read infrared fluorescence of the secondary antibodies. The Image Studio Lite software (LI-COR Biosciences; USA) was used to quantify target protein signal relative to the normalizer.

### Generation of human 3D engineered skeletal muscles

3D muscles were generated following established protocols.^74^^,^^77^ Briefly, 10^6^ human immortalized myoblasts were resuspended in 120 μL of a matrix composed of 3.5 mg/mL human fibrinogen (Tissucol Duo 500, Baxter; Sigma-Aldrich, F8630), 10% Matrigel (BD, 356230), and 3 U/mL thrombin (Biopur, 10-13-1104; Sigma-Aldrich, T7326). The hydrogel was polymerized between two polydimethylsiloxane (PDMS) posts (EHT Technologies, Hamburg) in an agarose mold for 2 h at 37°C. Following polymerization, constructs were cultured at 37°C in 5% CO_2_ in growth medium (PromoCell, C-23060) supplemented with 33 μg/μL aprotinin (Sigma, A3428) to inhibit fibrin degradation. To induce myogenic differentiation, muscles were transferred to differentiation medium (PromoCell, C-23061) supplemented with aprotinin 48 h after polymerization. Medium was refreshed every other day until day 14 post polymerization. 3D muscles for contractility assays were generated on the Optics11Life Cuore device as described in the specific section (functional assays in 3D engineered skeletal muscles). Although WT and DMD myoblasts used in this study were not isogenic, 3D muscles were generated simultaneously from a single batch of myoblasts for each genotype and differentiated in parallel in independent cell culture wells.

### Processing and immunostaining of 3D engineered skeletal muscles

3D muscles were fixed overnight at 4°C in PBS + 1% PFA (JI9943.K2, Thermo Scientific). Following fixation, samples were subjected to a sucrose gradient for cryoprotection, with sequential 16-h incubations in 10%, 15%, and 30% sucrose solutions (S0389, Sigma) at 4°C. The muscles were then embedded in Tissue-Tek OCT compound (4583, Sakura Finetek) on dry ice. 8-μm transverse sections were prepared using a cryostat (Leica) and mounted onto a glass slide. Sections were washed twice in PBS (P4417, Sigma), permeabilized with PBS containing 0.5% Triton X-100 (PBST) (T8787, Sigma), and blocked in PBST + 10% BSA for 1 h at RT. Primary antibodies diluted in 5% BSA (Sigma, A7906-100G) were applied overnight at 4°C. The next day, sections were washed in PBST, incubated for 2 h at RT with Hoechst 33342 (B2261, Sigma) and species-specific secondary antibodies, then washed again four times with PBST.^74^ Finally, the stained sections were mounted for downstream microscopic analysis.

### Functional assays in 3D engineered skeletal muscles

#### Calcium transients

Calcium transients assay was performed following established protocols.^77^ 3D muscles generated on PDMS posts were incubated in a 24-well plate with 2 mL of phenol red-free Opti-MEM basal medium containing Fluo-4AM (F14201, Thermo Scientific) for 30 min at 37°C. The muscles were then washed in Opti-MEM for 10 min before calcium transient assay. EHT carbon electrodes (EHT Technologies, Germany) were carefully positioned on either side of the muscle without direct contact and temporarily secured to the well using Blu Tack. The setup was placed on a Zeiss LSM 880 confocal microscope. Baseline fluorescence was recorded for 10 s without stimulation. Then, a pulse generator connected to the electrodes applied a series of electrical stimulations at 0.5 Hz: 30 s of stimulation at 5 V and 30 s at 10 V. Fluorescence excitation was performed at 488 nm and emission was collected at >530 nm. Time-lapse acquisition was carried out at 33 frames per second using a 10× objective, focusing on fields containing aligned myotubes. 10-V stimulation time-lapse images were exported and analyzed using the FiJi software. Mean fluorescence intensity over time was extracted by plotting the *z* axis profile, enabling quantitative assessment of intracellular calcium transients.

#### Contractility

Initial assessment of contractility (Figure S3K) was performed following established protocols.^77^ Briefly, 3D muscles generated on PDMS posts were placed in a 24-well plate with 2 mL of muscle differentiation medium. The plate was then positioned on an elevated holder above a Dino-Lite digital microscope camera (Dino-Lite Digital Microscopy, the Netherlands) for video acquisition. Carbon electrodes (EHT Technologies, Germany) were carefully positioned on either side of each muscle without direct contact and temporarily secured to the well using Blu Tack. Prior to electrical stimulation, a 10-s baseline recording was performed to assess spontaneous contractile activity. Electrical stimulation was then delivered via a pulse generator (0.5 Hz, 5–10 V). Muscle contractions were recorded as.avi files and analyzed using the MUSCLEMOTION plugin^117^ in ImageJ. For quantitative contraction assay (Figures 3G), 3D muscles were generated using 5 × 10^5^ cells in the Cuore device (OPTICS11 Life, the Netherlands).^118^ On day 10 of differentiation, the electrode board, consisting of carbon plate electrodes, was added to the Cuore Smartlid. This electrode board was connected to a pulse generator that delivered electrical stimulation to the muscles (twitch: 1 Hz, 12 V, 30 s. Tetanus: 50 Hz, 12 V, 30 s). Contractile force, measured in μN, was assessed using the Cuore software. Consistency of force measurements was assessed for both pre- and post-tetanic contraction, obtaining similar profiles.

### Mouse studies

Four-week-old male *mdx* mice were treated with AAV9 by tail-vein or intramuscular (TA) injection. A total of 1e13 vg of AAV9-CMV-Cas9 and AAV9-gRNA (1:5 ratio) was injected for intravenous study, and 3e11 vg were injected in the TA for intramuscular study. For histological and molecular analysis of mouse tissues, organs were collected immediately after animals were sacrificed by cervical dislocation, snap frozen in liquid-nitrogen-cooled isopentane, and stored at −80°C.

### Histological analysis of mouse organs

8-μm transverse cryosections of frozen TA, DIA, or HR muscles were prepared, air-dried, and stored at −80°C until further use. Mouse sections were subjected to hematoxylin and eosin (H&E) staining following previously described protocols.^106^ Whole-muscle sections were imaged using an AxioScan Z1 automated slide scanner (Zeiss, Germany) equipped with ZEN 2.6 SlideScan software and a Plan APO 10×/0.45 NA objective. The percentage of centrally nucleated fibers was quantified by analyzing H&E-stained images of entire muscle cross-sections. For fibrosis analysis, mouse muscle sections were stained with Sirius red as previously described.^119^ Quantification of fibrosis was performed with QuPath^120^ using the pixel classifier tool. A random-forest-method-based machine-learning model was trained using manually annotated ground truth regions, including areas labeled as “fibrosis,” “muscle,” and “blank” (regions without tissue or containing fat). For each section, the muscle-containing area was delineated using QuPath’s brush and wand tools while excluding regions with visible artifacts such as tissue folding. The trained classifier was then applied to the defined region, and areas corresponding to each class were quantified. Fibrosis ratio was calculated by dividing the total fibrosis area by the total area of the analyzed muscle section.

### Immunofluorescence staining of mouse organ transverse sections

Frozen transverse muscle sections were fixed in acetone for 10 min and blocked in M.O.M. (Mouse on Mouse) (BMK-2202, Vector Laboratories) for 30 min at RT. Sections were then incubated overnight at 4°C with primary antibodies: mouse anti-UTRN (1:50, sc-33700, Santa Cruz Biotechnology) and rat anti-laminin (1:50, sc-59854, Santa Cruz Biotechnology). The following day, sections were washed in 1× PBS and incubated for 1 h at RT with appropriate Alexa Fluor-conjugated secondary antibodies: goat anti-mouse IgG (1:500, A11013, Thermo Fisher) and goat anti-rat IgG (1:1,000, A11005, Thermo Fisher). Whole-muscle sections were then imaged using the AxioScan Z1 automated slide scanner (Zeiss, Germany), with ZEN 2.6 SlideScan software and a Plan APO 10×/0.45 NA objective. Each fiber was segmented thanks to the laminin-α2 labeling using the Morpholib plugin (Fiji software v2.0.0-rc/1.52p, Morpholib plugin v1.4.1). Fluorescence intensity was measured at the membrane across all channels. Negative conditions (no primary antibody) were used as controls.

For native GFP signal analysis, a portion of TA was fixed in 4% PFA and treated with sucrose before being cryopreserved, sectioned, and mounted on a glass slide with Fluoromount-G with DAPI (00-4959-52, Thermo Fisher Scientific). Acquisition of the native GFP signal on 8-μm transverse sections was performed using the AxioScan device.

### TA *in situ* contraction assay

The procedure was performed using the Aurora Scientific system with the Dynamic Muscle Control and Dynamic Muscle Analysis software (Aurora Scientific, Canada). Briefly, an analgesic was administered, followed by anesthesia 30 min later. The patellar tendon and the distal tendon of the TA were isolated, the latter being attached to a ring connected to a force detector. The sciatic nerve was also isolated and linked to a stimulation electrode. After measuring the baseline signal, a tetanic contraction was induced using the following parameters: 125 Hz, 300 ms. A single twitch was then performed to assess the force generated by a single stimulus. For each muscle (left and right TA), both maximal tetanic force and maximal twitch force were measured, normalized to muscle weight, and corrected by subtracting the baseline signal.

### Statistical analysis

All results were analyzed using GraphPad Prism version 7.00 for Windows (GraphPad Software, La Jolla, CA, USA; www.graphpad.com) or R (version 4.5.0). Normal distribution was assessed using Shapiro-Wilk normality test. Upon normal distribution, we performed a one-way analysis of variance (ANOVA) with *post hoc* tests to correct for multiple comparisons. For human *in vitro* qPCR data, fold change values were log transformed to meet model assumptions. For human *in vitro* western blot data, a different analysis was performed to account for high normalized signal intensity variability between biological replicates. Normalized fluorescence values were log transformed to meet model assumptions. To account for batch-to-batch variability in the experimental design, we employed a linear mixed-effects model using the R lme4 package (version 1.1-37). The model included treatment group (control, EBS, Let-7c) as a fixed effect and experimental batch as a random effect to account for the matched structure of the data. The model was specified as log(normalized fluorescence) ∼ group + (1|batch), where group represents the fixed effect of treatment and (1|batch) represents the random intercept for batch. Missing values were handled using restricted maximum likelihood (REML) estimation, which provides unbiased estimates under missing-at-random assumptions. Pairwise comparisons were performed using estimated marginal means with the emmeans package, and Tukey’s *post hoc* test. Model assumptions were verified by examining residual plots for homoscedasticity and Q-Q plots for normality of residuals and random effects.

All values are expressed as mean ± standard deviation of the mean (SD). “*n*” indicates the number of biological replicates for each group. Differences are considered statistically significant when *p* < 0.05.

## Data and code availability

The authors declare that all data supporting the findings of this study are available in the main text or the supplementary materials. The Guide-seq and Amplicon-seq data that support the findings of this study have been deposited (https://www.ncbi.nlm.nih.gov/sra/?term=PRJNA941216). Any other relevant data are available at reasonable request.

## Acknowledgments

We acknowledge the Genethon Vector Core Facility for their help with AAV production, the Genethon Functional Evaluation Facility for their help with mouse experimentation, the Genethon Histology Facility for their help with histology/immunofluorescence, the Genethon Imaging and Cytometry Core Facility for image analysis and technical support, and the Imagine Genomic Platform for Guide-seq and Amplicon-seq. We also acknowledge the CERFE for animal work. We acknowledge the MyoLine platform for immortalization of human cells, Myobank-AFM, and Dr. J.P. Concordet for Cas9 protein production (supported by ANR-II-INSB-0014). We thank members of the Therapeutic Genome Editing group for fruitful discussions and experimental help. We thank Guillaume Corre, Marine Rouillon, and Margaux Mombled for Guide-seq data analysis; Valentina Lionello for help and assistance with 3D muscle functional tests; and Bérangère Bertin for some AAV productions. We are grateful to Ile-de-France Region, to Conseil Départemental de l’Essonne (ASTRE), INSERM, and GIP Genopole, Evry for the purchase of the equipment.

This work was funded by the 10.13039/501100000780European Union (10.13039/100018693Horizon Europe project no. 101080690–MAGIC); the 10.13039/100014013UK Research and Innovation (UKRI) under the UK Government’s Horizon Europe funding guarantee grant nos. 10080927, 10079726, 10082354, and 10078461; the Swiss State Secretariat for Education, Research and Innovation (SERI) (www.magic-horizon.eu/; M.A. and F.S.T.); Paris Ile de France Region DIM Thérapie génique initiative (M.A. and M.R.); and grants from the 10.13039/501100001665Agence Nationale de la Recherche (HemoLen ANR-20-CE17-0016-0 [M.A.]; PEMGeT ANR-22-CE17-0028-02 [M.A.]; IRIS ANR--21-CE14-0063-03 [M.A.]; NEEDED ANR-24-CE18-3712-01 [M.A.]), 10.13039/501100007149Genopole (Programme ApogeeBio [S.G.]), 10.13039/501100004923AFM-Téléthon (M.A.), 10.13039/501100015760Inserm (M.A.), and 10.13039/100020987University of Évry Val d'Essonne (M.A. and P.G.). The authors also acknowledge funding from the 10.13039/501100000780European Union (EDITSCD grant 101057659, https://editscd.eu/ (M.A.); ERDERA grant 101156595 (M.A.); HISTOID grant 759108 (F.S.T.); DREAMS grant 101080229, https://dreamshorizon.eu/ (F.S.T.); and the 10.13039/501100000921European Cooperation in Science and Technology (COST) (GeneHumdi-CA21113 [M.A.]). Views and opinions expressed are those of the authors only and do not necessarily reflect those of the European Union or the HaDEA. Neither the European Union nor the granting authority can be held responsible for them.

## Author contributions

S.G. and M.A conceived the study. M.R., S.G., S.D., P.G., E.S., F.M., and F.A. contributed to the execution and/or analysis of the experiments. A.d.C. provided the Cas9 protein used for *in vitro* experiments. I.R. provided *mdx* mice for starting the colony. K.M. provided the myoblast cell lines used for *in vitro* experiments. G.R. helped with some AAV production. F.S.T and M.A. supervised the study. M.R., S.G., and M.A. wrote the original manuscript with input from S.D., F.S.T., and all other authors.

## Declaration of interests

S.G. and M.A. are inventors on a patent application related to this work filed by Genethon (PCT/EP2021/076882; 10243723, filed 29 September 2021). M.A. and F.S.T. are part of the Horizon Europe-UKRI-SERI “MAGIC” consortium (www.magic-horizon.eu), which has DMD as one of its target diseases and includes four private small and medium-sized enterprises (SME) partners. F.S.T. is inventor on a patent application on muscle tissue cultures (WO/2024/261261); he is academic principal investigator (PI) of a Crick-AstraZeneca partnership project, and his unrelated consultancies are managed by UCL Consultants.

## Supplemental information

Document S1. Figures S1–S6 and Tables S1 and S2

Document S2. Article plus supplemental information
