# Supplementary material for: CRISPR-Cas9-mediated upregulation of utrophin ameliorates Duchenne muscular dystrophy
Source: Mol Ther. 2026 Mar 24;34(7):3903–23. doi: 10.1016/j.ymthe.2026.03.025 (PMC13330077; doi:10.1016/j.ymthe.2026.03.025)
Supplement: Supplementary file 1 [file mmc1.pdf]

YMTHE, Volume *34*

Supplemental Information

CRISPR-Cas9-mediated upregulation of utrophin ameliorates Duchenne muscular dystrophy

Maëlle Ralu, Simon Guiraud, Sumitava Dastidar, Paola Galbiati, Emilia Sadaoui, Fetta Mazed, Fatima Amor, Anne de Cian, Isabelle Richard, Kamel Mamchaoui, Giuseppe Ronzitti, Francesco Saverio Tedesco, and Mario Amendola

A)

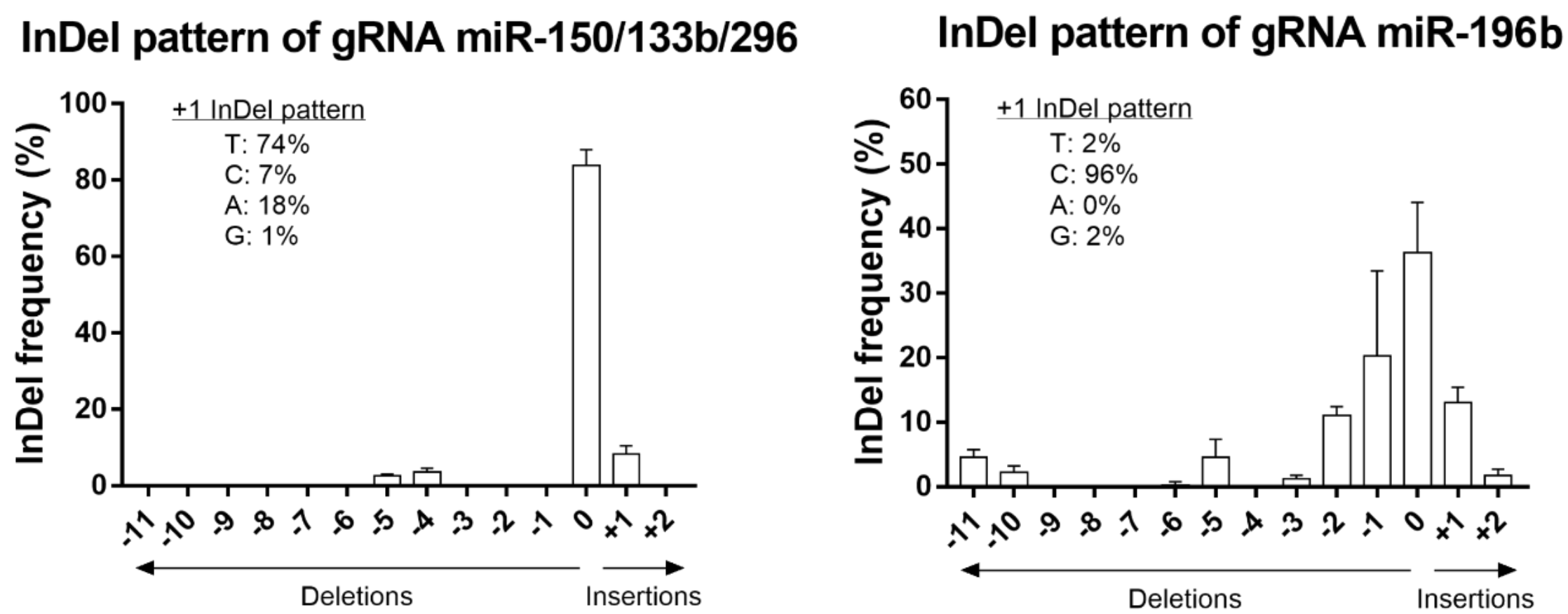

B) Off-targets in HEK-293T cells - gRNA Let-7c

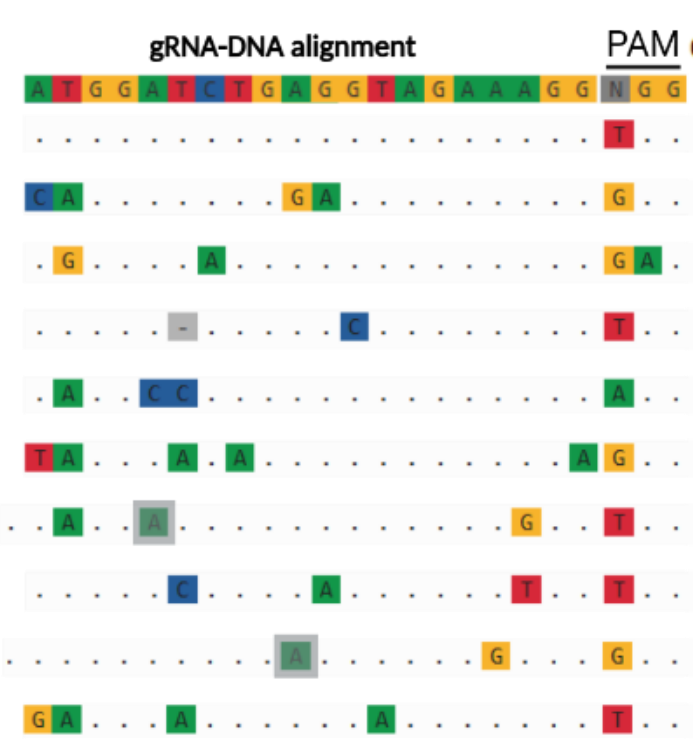

Genomic locus	Number of cells	Target	Gene ID	Position	Bulge
6:144852595	4590	On	UTRN	exon	-
22:31278943	1878	OFF	LIMK2	exon	-
1:151088396	816	OFF	GABPB2	intron	-
2:177025937	709	OFF	ENSG00000229337	intron	gRNA
X:69232729	496	OFF	-	-	-
2:175965830	443	OFF	LNPK	intron	-
X:51251180	220	OFF	LINC01284	intron	gDNA
X:11515139	168	OFF	ARHGAP6	intron	-
4:186702829	138	OFF	FAT1 / ENSG00000287169	intron / intron	gDNA
17:42774697	136	OFF	VPS25	exon	-

<u>Figure S1:</u> gRNA patterns and specificity.

A) *DNA InDel pattern in edited myoblasts. “+1 InDel pattern” indicates the frequency of each inserted nucleotide. N=4 biological replicates.* ***B)*** *Off-target analysis by Guide-seq in HEK 293T cells treated with gRNA Let-7c. The top 10 genomic sequences identified are listed. On the left, alignments of the gRNA to the genomic DNA sequences are shown. Perfect matches are represented by black dots and mismatches are represented by colored boxes. Grey boxes indicate bulges between the DNA and the gRNA sequence. On the right, information about each hit is indicated.*

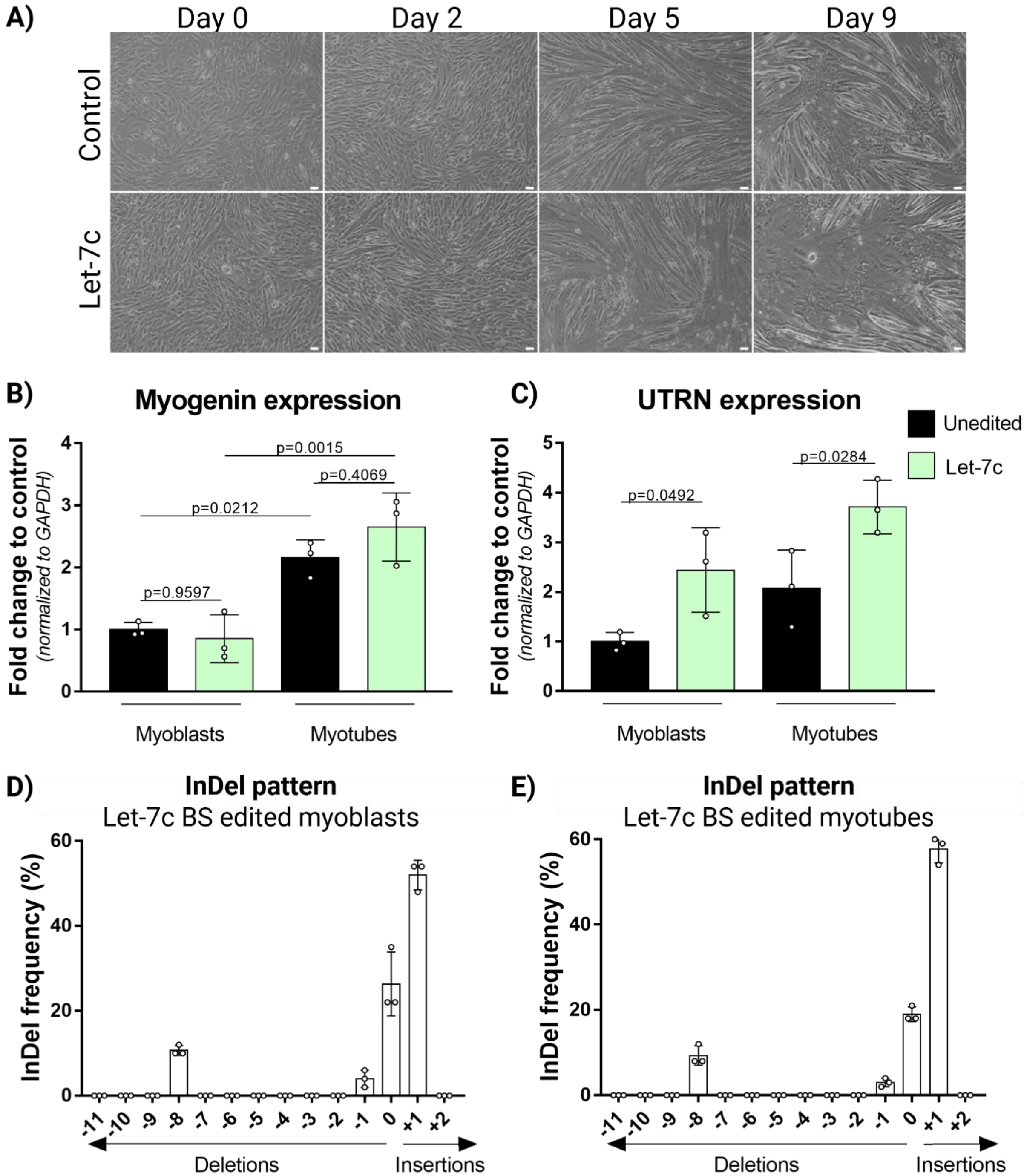

<u>Figure S2:</u> Let-7c BS disruption does not impair differentiation potential in hDMD myoblasts. ***A)*** *Representative brightfield images of control DMD or DMD-Let-7c BS edited myotubes across the myogenic differentiation process. Scale bar: 100 µm.* ***B)*** *Myogenin mRNA expression normalized to GAPDH and represented as fold change to unedited myoblasts.* ***C)***

UTRN mRNA expression normalized to GAPDH and represented as fold change to unedited myoblasts. ***D)*** *DNA InDel pattern in Let-7c BS edited myoblasts or* ***E)*** *myotubes.*

Each dot represents one biological replicate. N=3 independent differentiations per group. Bars represent the mean ± SD. Statistical analysis was performed using One-Way ANOVA with Tukey's (A) or Sidak's (B-C) post-hoc tests.

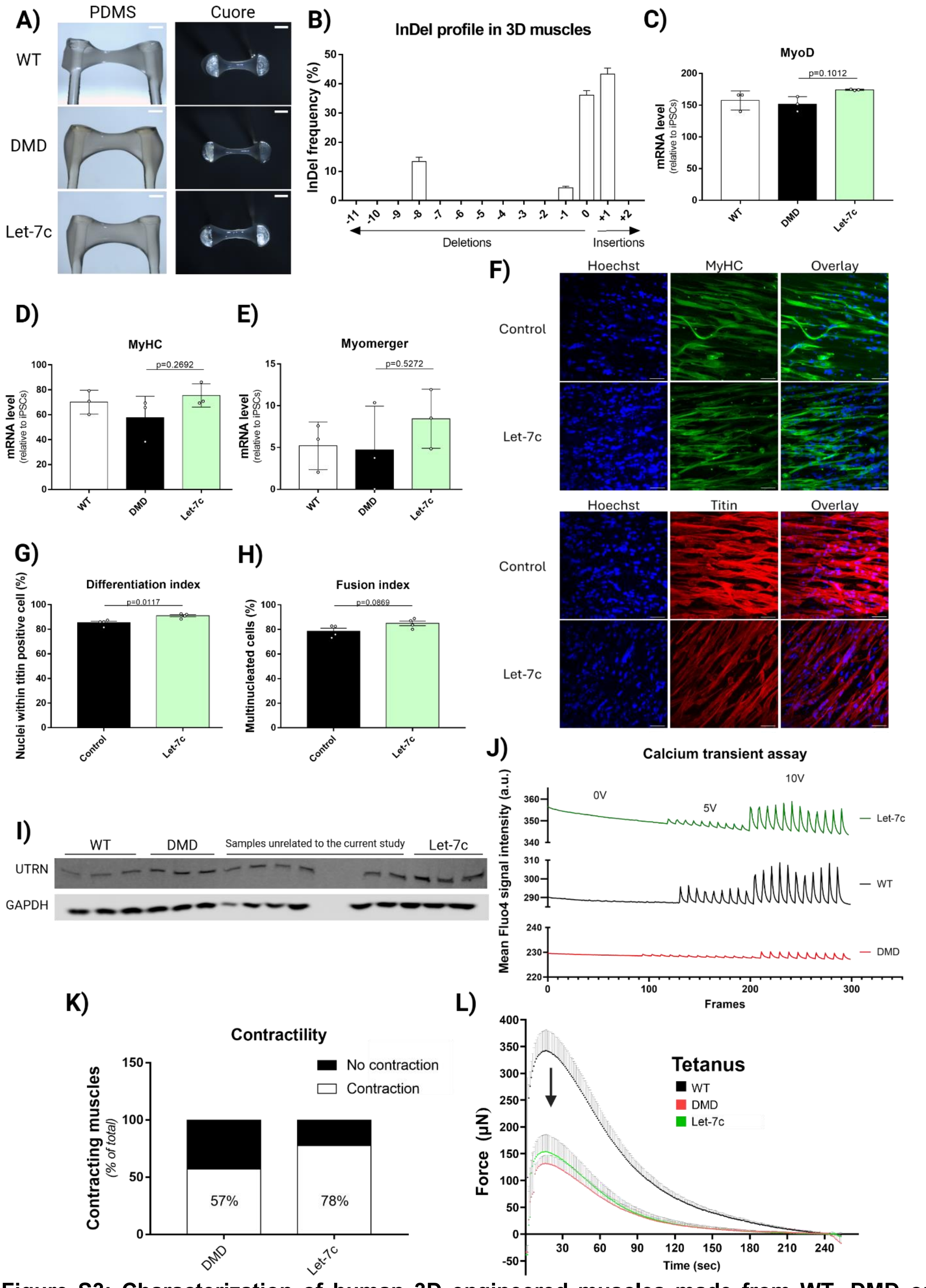

Figure S3: Characterization of human 3D engineered muscles made from WT, DMD or

CRISPR-edited DMD myoblasts. ***A)*** *Representative view of WT, DMD or DMD-Let-7c BS edited human 3D engineered muscles generated with polydimethylsiloxane (PDMS) posts (side view, 1e6 cells per muscle; details in Materials and Methods) or with the Cuore device (bottom view, 5e5 cells per muscle). PDMS-generated muscles were used for molecular, calcium and qualitative contractility assays. Cuore-generated muscles were used for molecular and quantitative contractility assays. Scale bar: 1000 µm.* ***B)*** *DNA InDel pattern in 3D muscles made from DMD-Let-7c BS edited myoblasts. n=3 per group.* ***C-E)*** *MyoD (C), myosin heavy chain (MyHC) (D) and Myomerger (E) mRNA expression normalized to GAPDH in WT, DMD or DMD-Let-7c BS edited 3D muscles relative to iPSCs, which do not express these markers. n=3 per group.* ***F)*** *Representative images of MyHC and Titin immunofluorescence staining of whole 3D muscles. Scale bar: 50 µm.* ***G)*** *Differentiation index (percentage of nuclei in Titin positive cells (any number of nuclei / total number of nuclei)) of control DMD or DMD-Let-7c BS edited 3D muscles. n=4 per group.* ***H)*** *Fusion index (percentage of myotubes with more than one nucleus) of control DMD or DMD-Let-7c BS edited 3D muscles. n=4 per group.* ***I)*** *Original unedited image of Western blot presented in Figure 3.D. Samples unrelated to the current study and removed from Figure 3.D are indicated.* ***J)*** *Representative results of a calcium transients assay performed in WT, DMD or DMD-Let-7c BS edited 3D muscles.* ***K)*** *Percentage of PDMS-generated muscles which contract upon electrical stimulation.* ***L)*** *Representative result of a tetanic contraction assay performed using the Cuore device. The arrow highlights the difference between Let-7c BS edited and DMD samples.*

Each dot represents one muscle. n indicates the number of whole, individual 3D muscles per group. Bars represent the mean ± SD. Statistical analysis was performed using One-Way ANOVA with Tukey's post-hoc test (C,D,E), unpaired t-test (G,H), or RM-Two-Way ANOVA with Sidak's post-hoc test (L).

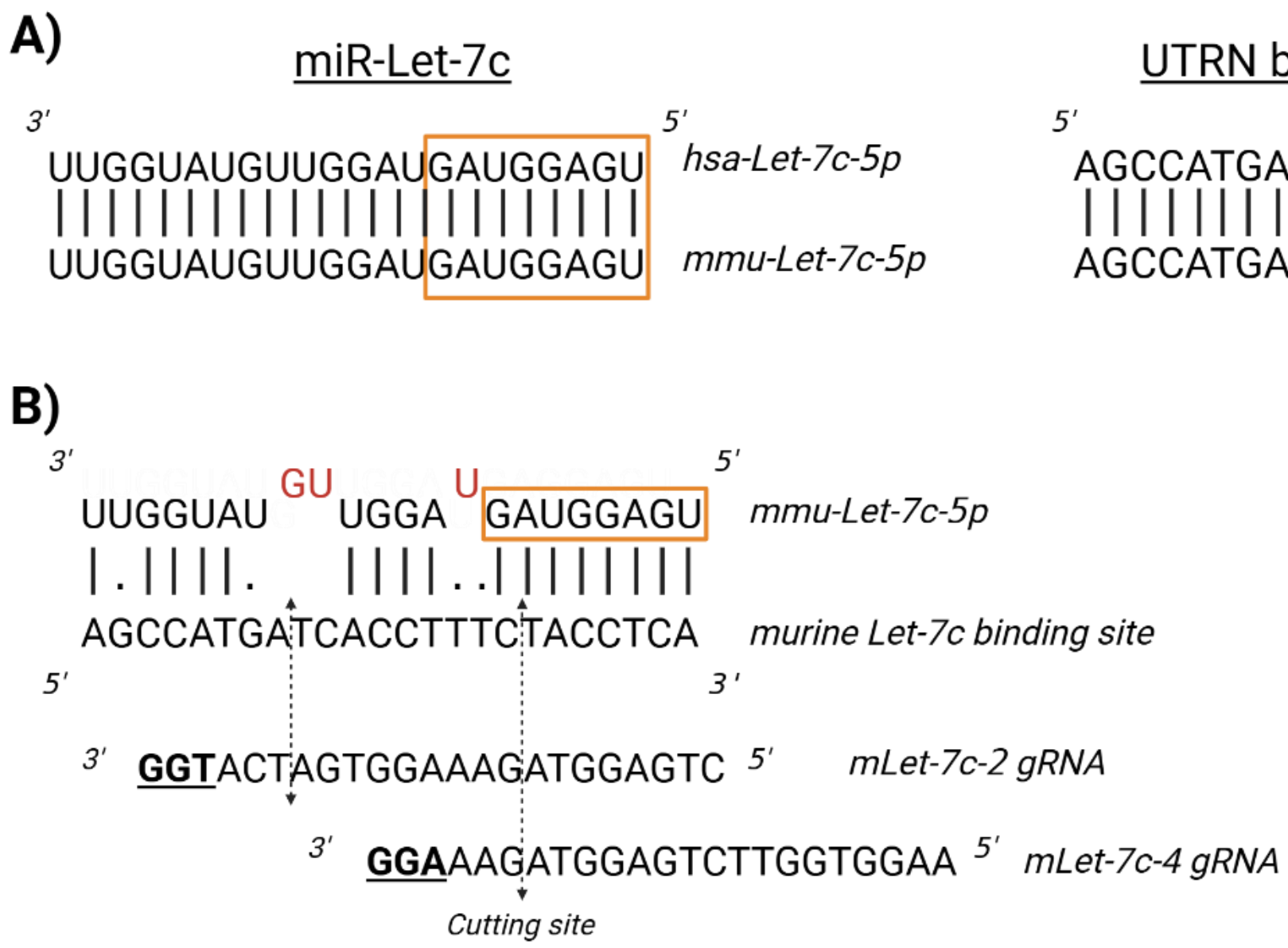

<u>Figure S4:</u> Evolutionary conservation of miR-Let-7c and its binding site in the UTRN sequence. A) *Alignments of the human (top) and mouse (bottom) sequences for miR-Let-7c (left) and its binding site in the 3'UTR region of UTRN (right). The seed sequence of the miRNA, essential for target recognition, is framed in orange. Differing nucleotides are highlighted in red. The miRNA sequence is fully conserved and its binding site in UTRN only differs by one nucleotide.* ***B)*** *Alignment of murine mmu-Let-7c-5p miR with its UTRN target and gRNA design for site disruption. The seed sequence of the miRNA is framed in orange. PAM sequences are shown in bold underlined font. Cutting sites for each gRNA are indicated by black dotted lines.*

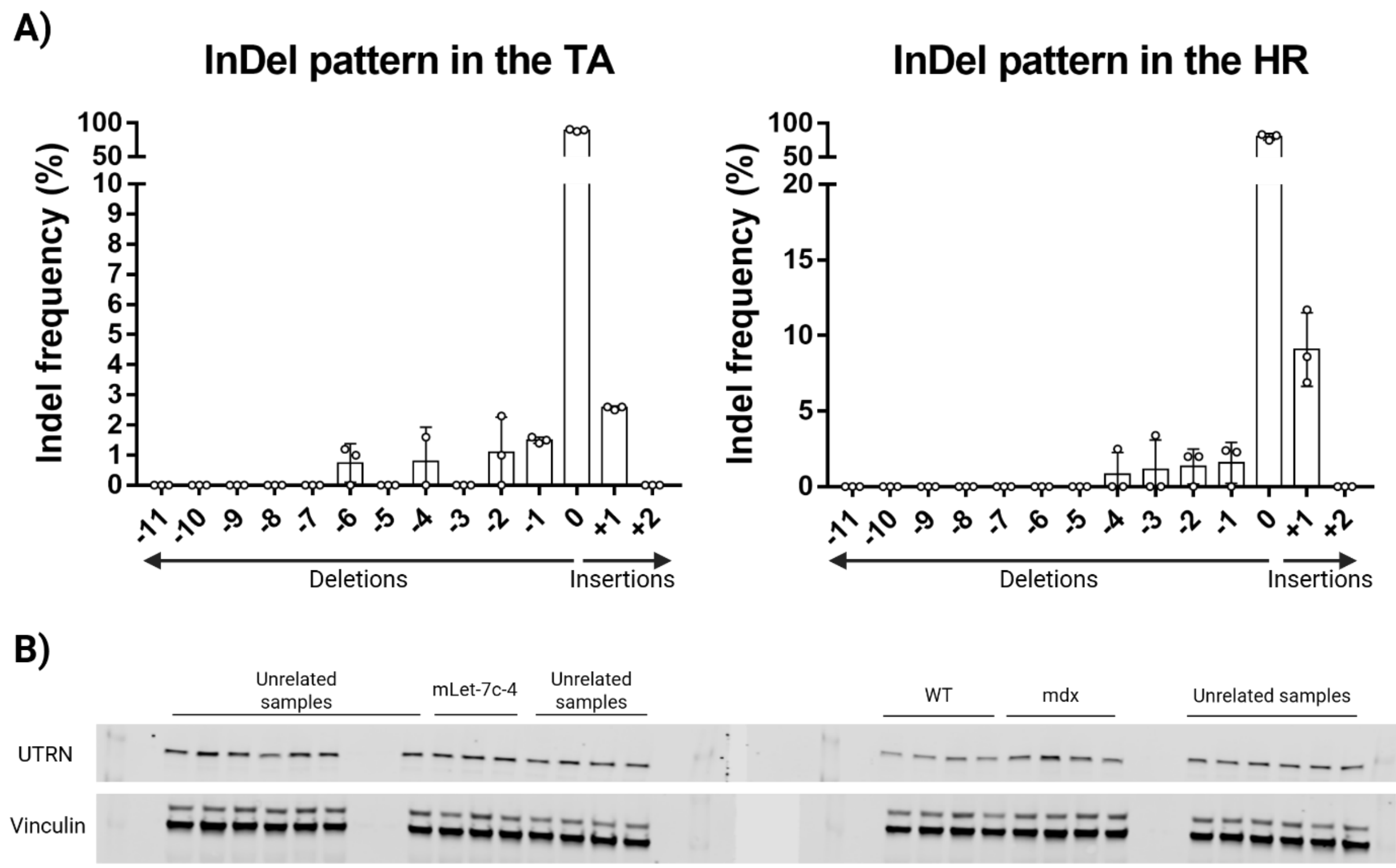

<u>Figure S5:</u> InDel pattern and protein level of *mdx* mice treated with injection of rAAV9-Cas9 and rAAV9-gmLet-7c-4. ***A)*** *DNA InDel pattern in the tibialis anterior (TA) and heart (HR) of mice treated by systemic injection. Each dot represents one mouse. N=3 mice per group. Bars represent the mean ± SD.* ***B)*** *Original unedited image of Western blot presented in Figure 5.F (mice treated by local injection). Samples unrelated to the current study and removed from Figure 5.F are indicated. WT/mdx and mLet-7c-4 samples were loaded on separate gels but run, transferred, blocked, stained and revealed simultaneously, using the same equipment and solutions to ensure consistency.*

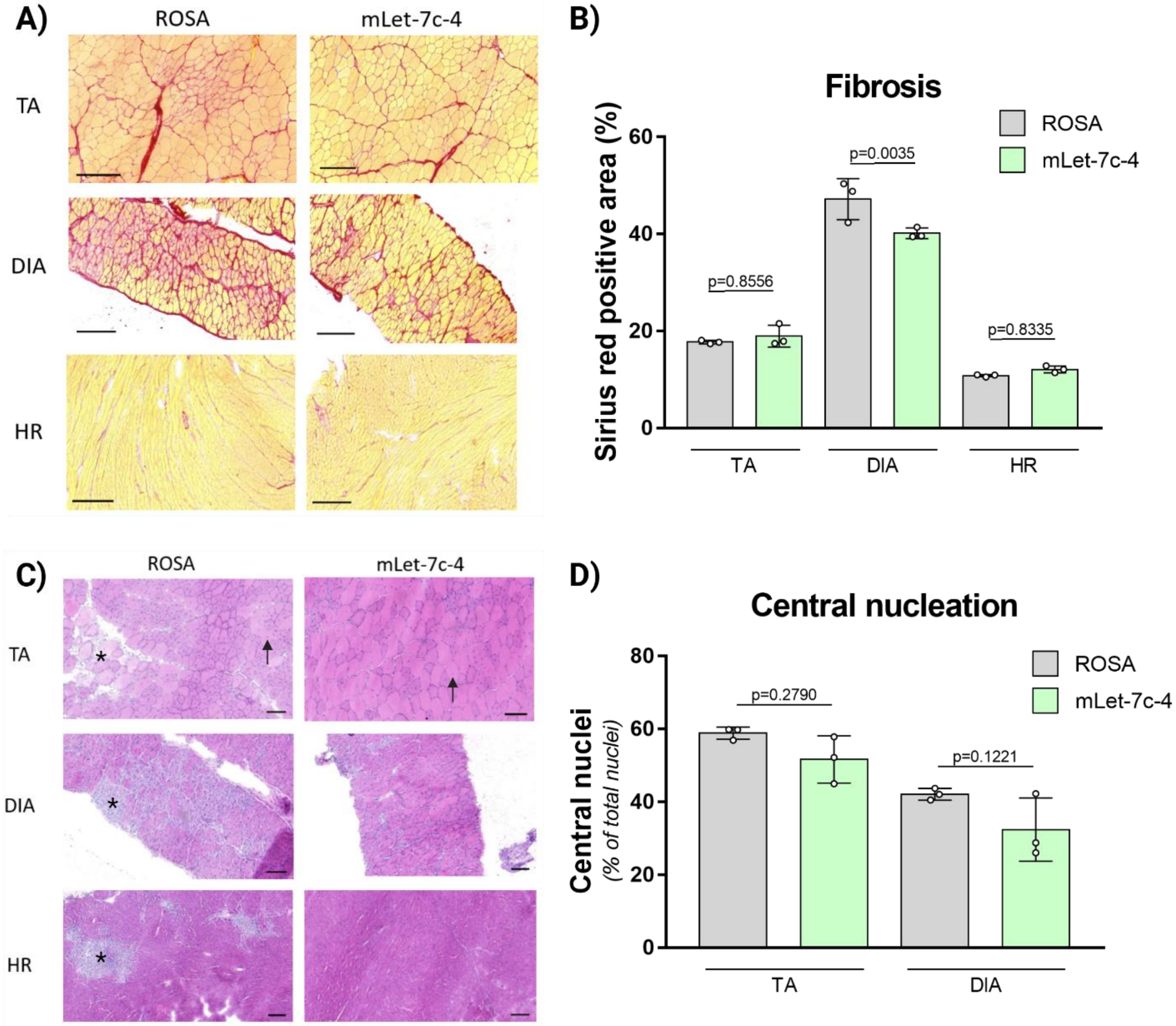

<u>Figure S6:</u> Systemic AAV9 delivery of Cas9 and Let-7c BS gRNA ameliorates several histological parameters in *mdx* mice. ***A)*** *Representative images of Sirius red stainings of tibialis anterior (TA), diaphragm (DIA) and heart (HR) transverse sections. Scale bar: 200µm.* ***B)*** *Quantification of Sirius red positive area (collagen deposition).* ***C)*** *Representative images of hematoxylin and eosin staining of TA, DIA and HR transverse sections. Regions of necrosis and infiltration are indicated by black stars. Examples of centrally nucleated fibers are indicated by a black arrow. Scale bar: 100µm.* ***D)*** *Quantification of centrally nucleated fibers.*

Each dot represents one mouse. N=3 mice per group. Bars represent the mean ± SD. Statistical analysis was performed using One-Way ANOVA with Sidak's post-hoc test.

<u>Table S1:</u> gRNA sequences.

gRNA name	Target site	gRNA sequence (5'-3')	PAM site	Strand
miR-150/133b/296	miR 150/133b/296-5p BS	TTATTTTAGAATAGGTTGGG	TGG	+
miR-196b	miR-196b BS	GTGCTTTCTTGGGTATGACA	TGG	-
Let-7c	Human Let-7c BS	ATGGATCTGAGGTAGAAAGG	TGG	-
mLet-7c-2	Mouse Let-7c BS	CTGAGGTAGAAAGGTGATCA	TGG	-
mLet-7c-4	Mouse Let-7c BS	AAGGTGGTTCTGAGGTAGAA	AGG	-
EBS	Human EBS	TCTTCCGGAACAAAGTTGCT	GGG	+
Control	HBA (human control)	GGGTTCTCTCTGAGTCTGTG	GGG	-
ROSA	ROSA (mouse control)	CTCCAGTCTTTCTAGAAGAT	GGG	-

<u>Table S2:</u> PCR primer sequences.

Target	Forward primer sequence	Reverse primer sequence
Human UTRN 3'UTR	TGGTACCTCCACCTACATCTT	GGGTATGACATGGCCTGATAAA
Human EBS	CACGCACGGTTCACTCT	CAACTTTGCCAAACGCTACA
Mouse Let-7c BS	GGTTCTCTTTCACTGGGATCTG	CCGGGAGTTTCTGTTGATCTT
HBA	TATCGCCAGAGGGAAAGGGA	CTTGAAGTTGACCGGGTCCA
ROSA	GCAGAAGGAGCGGGAGAAAT	GCTCAGTTGGGCTGTTTTGG
